# HTT-OMNI: A Web-based Platform for Huntingtin Interaction Exploration and Multi-omics Data Integration

**DOI:** 10.1016/j.mcpro.2022.100275

**Published:** 2022-08-03

**Authors:** Michelle A. Kennedy, Todd M. Greco, Bokai Song, Ileana M. Cristea

**Affiliations:** Department of Molecular Biology, Princeton University, Lewis Thomas Laboratory, Princeton, New Jersey, USA

**Keywords:** protein–protein interactions, huntingtin, Huntington's disease, computational platform, multiomics, HD, Huntington’s disease, HINT, Huntingtin protein-protein interaction database, HIP, Huntingtin-interacting protein, *Htt*, Huntingtin gene, HTT, Huntingtin protein, HTT-OMNI, Huntingtin omics and network integration viewer, MS, mass spectrometry, PPI, protein–protein interaction, snRNA-seq, single-nucleus RNA sequencing

## Abstract

Huntington's disease (HD) is a progressive neurological disorder that is caused by polyglutamine expansion of the huntingtin (HTT) protein. With the hope to uncover key modifiers of disease, a focus of the field of HD research has been on characterizing HTT-interacting proteins (HIPs) and the effect of the HTT polyglutamine expansion on the cellular omics landscape. However, while hundreds of studies have uncovered over 3000 potential HIPs to date, a means to interrogate these complementary interaction and omics datasets does not exist. The lack of a unified platform for exploring this breadth of potential HIPs and associated omics data represents a substantial barrier toward understanding the impact of HTT polyQ expansion and identifying interactions proximal to HD pathogenesis. Here, we describe the development of a web-based platform called HTT-OMNI (HTT OMics and Network Integration). This application facilitates the visualization and exploration of ∼3400 potential HTT interactors (from the HINT database) and their associated polyQ-dependent omics measurements, such as transcriptome and proteome abundances. Additionally, HTT-OMNI allows for the integration of user-generated datasets with existing HIPs and omic measurements. We first demonstrate the utility of HTT-OMNI for filtering existing HTT PPIs based on a variety of experimental metadata parameters, highlighting its capacity to select for HIPs detected in specific model organisms and tissues. Next, we leverage our application to visualize the relationships between HTT PPIs, genetic disease modifiers, and their multiomic landscape. Finally, we generate and analyze a previously unreported dataset of HTT PPIs, aimed at defining tissue-specific HTT interactions and the polyQ-dependent modulation of their relative stabilities in the cortex and striatum of HD mouse models.

## Highlights

• HTT-OMNI facilitates the exploration of huntingtin (HTT) PPIs and multi-omic data.
• The platform allows for the analysis of both existing studies and user-uploaded data.
• HTT-OMNI enables filtering of PPIs *via* metadata (model organism, method, etc.).
• Analysis of tissue-specific HTT interactomes in mouse models of Huntington’s disease.
• Relative stabilities of HTT PPIs in the cortex differ from those in the striatum.

## Graphical Abstract

**Figure:**
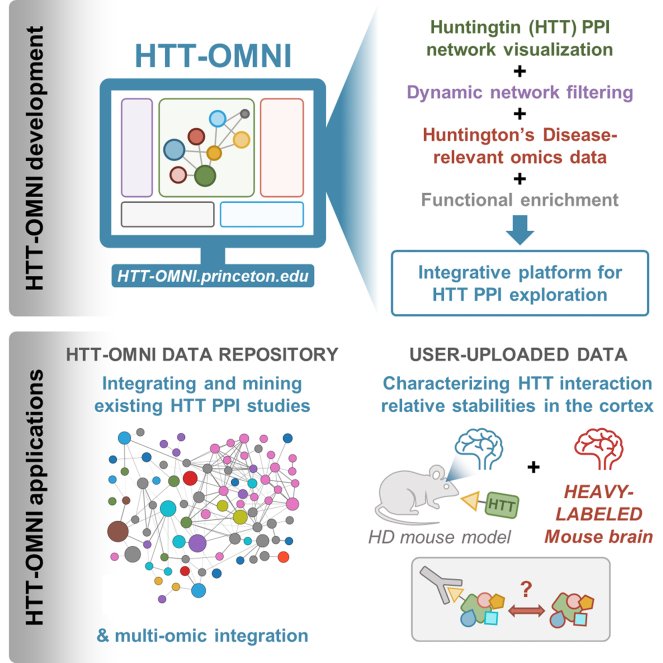

## In Brief

Kennedy *et al.* develop HTT-OMNI, a web-based platform for the visualization and analysis of huntingtin (HTT) PPIs and multi-omic data integration. They demonstrate the utility of HTT-OMNI for analyzing existing, as well as user-uploaded, datasets.

Huntington’s disease (HD) is an inherited neurodegenerative disorder characterized by progressive increases in involuntary motor movements, cognitive impairment, and behavioral abnormalities (1). HD is caused by a trinucleotide CAG repeat expansion in exon 1 of the huntingtin (*Htt*) gene (2). Although the underlying genetic mechanisms of HD pathogenesis are relatively well understood, the molecular, cellular, and systemic mechanisms that contribute to HD progression remain unknown. Furthermore, functions of the wild-type huntingtin protein (HTT) are still being uncovered in diverse cellular processes, including autophagy, endocytosis, vesicular trafficking, synaptic transmission, mitochondria homeostasis, transcription/translation (3), and more recently, DNA repair (4). Given that the disruption and dysregulation of protein interactions is known to underlie many human diseases (5, 6), efforts to understand the function of wild-type and mutant HTT in HD model systems have focused, in part, on characterizing HTT protein–protein interactions (PPIs). Over the last 25 years, >230 studies have characterized HTT PPIs, also referred to as huntingtin-interacting proteins (HIPs), using a plethora of different model systems, experimental techniques, and perturbations, prompting the curation of a huntingtin protein-protein interaction (HINT) database and delivery in an online data portal, HDinHD (7). The latest curation of the HINT database contains >9000 PPI reports corresponding to >3000 unique human orthologs. Reconciling the breadth of these interactions represents a substantial barrier toward understanding how *Htt* gene variants and polyglutamine expansion influence age of onset and disease progression in HD.

In addition to uncovering HIPs, another major focus of recent HD research has been the characterization of transcriptome and proteome changes in healthy and diseased states (*e.g.*, upon polyQ expansion) using mouse models. Meaningful integration of this information with the vast number of potential HIPs is challenging. Presently, various tools are available that enable researchers to explore these datasets. For example, the HDinHD portal (7) (hdinhd.org), curated by the CHDI Foundation and its academic and commercial partners, provides individual tools that facilitate either the interrogation of HTT PPI datasets (*via* HD Explorer) or polyQ-dependent measurements of RNA and protein levels (*via* ASViewer or HD Proteome Base). Another tool, HDNetDB (8), integrates molecular interactions with many HD-relevant datasets to allow researchers to visualize and prioritize relevant HTT targets and gene networks. However, HDNetDB is no longer available (9), and this tool only leveraged a subset of HTT interaction studies as it likely preceded the initial release of the curated HINT database. As such, simultaneous analysis of HTT-interacting proteins and their respective omics data is not currently possible with available tools.

To address these challenges, here, we describe the development of HTT-OMNI, a web-based platform for HIP visualization, exploration, and multi-omic integration. Within this paper, we (1) describe the development of the HTT-OMNI tool, (2) demonstrate its utility for visualizing and analyzing existing HTT PPI datasets, and (3) show how HTT-OMNI can be leveraged to examine a user-generated dataset. For this final purpose, as a proof of concept, we characterized alterations in HTT protein interactions in the cortex of HD mouse models. Specifically, we profiled HIPs in the cortex of an HTT Q140 knock-in HD and normal (HTT Q20) mouse using complementary label-free and isotope-labeled IP-MS approaches. While the striatum brain region shows the most striking neuropathological consequences of HD (10), impaired neuronal communication is observed between cells in the striatum (medium spiny neurons) and cortex (pyramidal neurons), particularly in early stages of the disease (11). Therefore, comparisons of PPI characteristics between the cortex and striatum of HD mouse models provide insights into cellular functions or pathways that may be common and tissue specific. Ultimately, HTT-OMNI summarizes and integrates known HTT PPIs with Q-dependent transcriptome and proteome measurements, providing an all-in-one exploratory platform that facilitates the prioritization of target genes that may contribute to HD pathogenesis. Our tool is freely available online at http://htt-omni.princeton.edu:5006/.

## Experimental Procedures

### Generation of HINTomics Database

A combined huntingtin protein interaction and HD omics database was generated as follows. **STEP I**: An existing catalogue of documented HIPs (HINT database) was downloaded in Excel format from the “Curated HD Datasets” section of the website HDinHD.org (2022_03), which contained 9951 unique HTT PPI observations compiled from 239 studies. **STEP II**: The HINT database was supplemented with two recent PPI studies (12, 13) that added an additional 388 entries to HINT (supplemental Table S1). Nonhuman gene symbols were mapped to their nearest human orthologs using the dbOrtho function of bioDBnet (14) or the DIOPT Ortholog Finder (15) (ver. 9.1). **STEP III**: A database of omics targets (genes/proteins) was assembled that were measured by proteomic and/or transcriptomic analysis of the allelic series HD mouse models (16). Processed transcriptome (mRNA-seq) and proteome (LC-MS/MS) datasets were obtained from the William Yang group, based on the study by Langfelder *et al*. (16), and from the “Mouse Allelic Series” section of HDinHD.org (7). Relative mRNA expression and protein abundances were collected from the striatum, cortex, cerebellum, hippocampus, heart, and liver tissues for Q50, Q80, Q92, Q111, Q140, and Q175 vs Q20 (control) mice at 2 (except for Q50), 6, and 10 months of age. The Q20 knock-in mouse was used as control, given that it is within the range of polyQ-lengths for the nonmutant huntingtin allele in the human population (2). For the HTT-OMNI application, replicate measurements for male and female were averaged. Nonhuman gene symbols were mapped to their nearest human orthologs as described in STEP II above (supplemental Table S2). **STEP IV**: For targets with bulk proteome and/or transcriptome measurements, values were annotated by single-nuclei RNA-seq relative expression in the striatum of Htt^Q175/+^ vs WT mice (17) and single-cell RNA-seq fractional expression in wild-type mouse striatum (18). **STEP V**: From the consolidated HD omics database (STEPS III and IV), we used the HINT database (STEP II) to annotate targets that were positive HIPs in at least one study. Note, any targets without a human ortholog were excluded from HINTomics database. In total, the human-centric HINTomics database contained 15,399 nonredundant entries (by gene ID), of which 3844 have been observed as HIPs in at least one study (n = 241).

### External Published Datasets and Localization Annotations

Mouse gene symbols were obtained from supplemental Table S14 of the study by Langfelder *et al*. 2016 that were differential at both RNA and protein levels by continuous Q statistical analysis (16). Mouse gene symbols were obtained from supplemental Tables S2 and S3 of the study by Wertz *et al*. 2020 that were identified as candidate HD modifiers from zQ175 HD mice, but were not found to be neuronal essential genes in the wild-type mouse striatum (19). For all datasets, mouse gene symbols were mapped to their nearest human homologs. Genes without a human homolog were excluded from the analysis. When applicable, the subcellular localizations of targets were determined stepwise by sourcing annotations from UniProt (20) and the Human Protein Atlas (21) and then manually assessed for assignment quality. Proteins that had multiple localizations were represented as being localized to multiple compartments (*e.g.*, cytoplasm and nucleus) so that this information was retained. These curated localization annotations were then uploaded to HTT-OMNI as a custom categorical annotation using the header flag “QUANT_localization.”

### Network Structure Development

HIPs were sourced from the assembled HINTomics database (see “Curation of HINTomics database”) to define the potential nodes available to HTT-OMNI for network generation. Since the HINT database contains extensive annotations (>70 columns), we prioritized a subset of these columns (model, model_organism, tissue, htt_length, common_name, cell_culture_comment, detection_method, and study_identifier) to be available for data filtering. Only interactions annotated as confirmed interactions (*e.g.*, marked with “Y” in the “interaction result” column) were included in HTT-OMNI. Note that the model and model_organism columns were partially redundant, so we combined these two columns into a single annotation “model_species.” Edges between nodes were downloaded from STRINGdb (22) using the Entrez Gene ID of each node as an identifier to query the protein.aliases.v11.5.txt.gz and protein.links.v11.5.txt.gz files available for download on the STRINGdb website.

### Application of Network Filters

To filter HTT interactions based on the aforementioned data filters, we applied the given filter conditions at the interaction level (*e.g.*, on the raw HINT data). This allows the user to specifically select HTT interactions derived from individual experiments that fit the filtering criteria. The options selected under each individual filter can be applied with one of three logical operators (AND, OR, or NOT), which are applied to all selected filter options selected for a given filter. For example, if “Mice” and “Human” have been selected with an “AND” logical operator under the “Model (organism)” filter, all HTT-interacting proteins that have been identified at least once in mice AND at least once in humans will be returned. For the sake of simplicity, the logical operator applied to different filters is always treated as “AND.” For example, if “Mice” is selected under the “Model (organism)” filter and “brain” is selected under the “Tissue” filter, all HTT-interacting proteins identified in experiments in mice brain tissues will be returned.

### Enrichment Analysis Using PANTHER

Enrichment analysis is performed using the PANTHER (23) database overrepresentation enrichment API using all genes in the HINTomics database as a background gene list. Statistical significance is determined using Fisher’s exact test with a false discovery rate correction. The annotation set that is queried is determined *via* user selection (GO biological process, GO molecular function, GO cellular component, GO SLIM molecular function, GO SLIM biological process, GO SLIM cellular component, Panther pathways, or Reactome pathways).

### Programs and Software

HTT-OMNI uses the following Python packages: Panel (version 0.13.0; dashboard development), HoloViews (version 1.14.8; interactive plotting), Pandas (version 1.4.2; data manipulation), Networkx (version 2.7.1; network creation), Requests (version 2.27.1; REST API querying of PANTHER), and Conda (version 4.12.0; environment management). An environment.yml file is available on GitHub to reproduce the Conda environment used in this study and to run HTT-OMNI locally. Figures were made in Microsoft PowerPoint.

### Mouse Strains

The huntingtin N-terminal Flag-tagged control (Htt3xFlagQ20/+) and HD knock-in (Htt3xFlagQ140/+) male and female mice are congenic in the C57BL/6J background. These mice were obtained from the group of Scott Zeitlin (University of Virginia School of Medicine), as in the study by Greco et al (12). Mice were housed in a humidity- and temperature-controlled room with 12-h light–dark schedule. Food and water were provided ad libitum. All experimental procedures were approved by the University of Virginia (UVA) Institutional Animal Care and Use guidelines, and precautions were taken to minimize stress and the number of animals used. The UVA is fully accredited by the Association for Assessment and Accreditation of Laboratory Animal Care (AALAC), and the university has a Public Health Service (PHS) Assurance on file with the Office of Laboratory Animal Welfare (PHS Assurance # A33245–01). Brains were harvested from male and female 2- and 10-month-old mice following isoflurane anesthesia and cervical dislocation. Brains were cut in half sagittally and immediately frozen in 2-methylbutane pre-equilibrated in dry ice. Cortices were isolated in a Petri dish on ice and placed immediately in 1.5-ml tubes prechilled in dry ice. Tissues that were used in paired control and experimental immunoaffinity purifications within the same biological replicate were sex matched.

### Experimental Design and Statistical Rationale

Label-free FLAG IP-MS experiments were performed from cortical tissues of Htt3xFlagQ20 and Htt3xFlagQ140 mice at 2 months and 10 months age, in three biological replicates for each genotype-age, including parallel IgG controls samples, also performed in three biological replicates. Isotope-labeled IP-MS experiments were performed in duplicate experiments. Overall, these numbers of replicates are influenced by practical sources of tissue availability in HD mouse model and supply of heavy-labeled mouse brain tissues. Overall, they are adequate for assignment of significant/differential interactions as we employed both statistical testing (student’s *t* test) and a strict fold-change difference threshold (FC ≥ 2.0).

### HTT Immunoaffinity Purification-Mass Spectrometry Analysis From the Mouse Cortex

Htt3xFlag IP-MS experiments in cortical tissues were performed as previously described (12) except the mass spectrometry analysis was conducted on a Q Exactive HF mass spectrometer (Thermofisher Scientific). Frozen tissues were removed from −80 °C and thawed on ice. Tissues were transferred to a precooled Potter-Elvehjem tissue grinder, lysed in 4 ml of lysis buffer (1xTBT buffer, 1% Triton X100, 150 mM NaCl, 2× Halt Protease and Phosphatase Inhibitors Cocktail, 100U/ml universal nuclease) with 2 × 10 strokes, incubated on ice for 5 min, and transfered to precooled 2 × 2-ml Eppendorf tubes. The insoluble material was removed by centrifugation (20,000*g* at 4 °C for 10 min). The supernatant was diluted to 10 ml with lysis buffer, and 2 × 4.5-ml aliquots were used for anti-FLAG and control IgG IPs in 5-ml Lo-bind tubes.

Anti-FLAG and nonspecific IgG antibodies were conjugated to Protein A/G magnetic beads (12 μg of antibodies for each 30 μl of bead slurry). The unconjugated beads were washed 3 × 500 μl of 1× TBT and then incubated with antibody for 1 h at 4 °C. After conjugation, beads were washed 2 × 500 μl of in lysis buffer. The IPs were carried out in biological triplicate using 30 μl of antibody-conjugated beads/IP, which were added directly to the supernatant of the lysed tissue and incubated for 1 h at 4 °C. The beads were collected on magnets and washed with lysis buffer (3 × 500 μl) and water (2 × 500 μl). Captured proteins were eluted in 1× TEL buffer (50 μl) heated at 70 °C for 10 min with brief vortexing and recovered by magnetic separation. Eluted proteins were reduced and alkylated with Tris(2-carboxyethyl)phosphine hydrochloride (0.5 μl of 500 mM TCEP) and chloroacetamide (1.5 μl of 500 mM), respectively, by heating at 70 °C for 20 min.

Filter-aided sample preparation was used to perform protease digestion of the samples using trypsin, followed by StageTip desalting and peptide fractionation (n = 3 fractions) as previously described (24, 25). Peptide fractions were suspended in 1% FA/1% ACN in a final volume of 5 μl. Desalted peptides (2 μl) were analyzed by data-dependent LC-MS/MS on a Q Exactive HF Hybrid Quadrupole-Orbitrap (QE) mass spectrometer (ThermoFisher Scientific).

### Metabolic Labeling Immunoaffinity Purification of 3×FLAG-Htt

^13^C_6_-lysine (≥97% enriched)-labeled mouse whole brain tissues collected at 2 months of age was purchased from a commercial source (MT-LYSC6-MB-PK, Cambridge Isotopes) for use in metabolic labeled IP-MS experiments as labeled cortical tissues were not practical to produce. The reported extent of ^13^C protein enrichment in these samples has been previously validated by whole-proteome analysis of heavy-labeled tissue (12). Immunoaffinity purification of 3xFLAG-Htt in metabolic labeled IP-MS experiments was performed similar to the label-free IP-MS experiments above and as in the study by Greco et al (12), except the cortical lysates (4.5 ml) were mixed with an equal protein amount (w:w) of sex- and age-matched ^13^C_6_-lysine labeled brain lysate extracted from the whole brain.

### Informatics Analysis of IP-MS Experiments

Raw instrument files were analyzed in Proteome Discoverer (v2.4.0.305), which recalibrated precursor ion masses, extracted MS/MS spectra, and performed a database search with Sequest HT (v1.17) to assign peptide spectrum matches (PSMs) against a mouse protein sequence database appended with common contaminants and concatenated with reverse entries for calculation of FDR thresholds (UniProt reviewed entries, downloaded 2017–07, 30,060 sequences). The database search was performed with the following settings: full trypsin cleavage specificity, allowing for up to two missed cleavages, a precursor and fragment ion match tolerance of 4 ppm and 20 ppm, respectively, fixed modifications of carbamidomethylation of cysteine, and variable modifications of oxidized methionine, deamidation of asparagine, and loss of methionine plus acetylation of the protein N-terminus. For isotope-labeled experiments, an additional variable modification of heavy ^13^C_6_-lysine was specified. Percolator (v3.02.1) was used to calculate PSM q-values for local FDR estimates. Search results were assembled using a consensus report that leveraged q-values to control global FDR to ≤1% at the spectra, peptide, and protein levels. Specific *versus* nonspecific interactions were determined with Significance Analysis of INTeractome (SAINT) (26) on the REPRINT server (https://reprint-apms.org/?q=reprint-home) using spectral counts as input from bait (Htt3xFlag) and control (IgG) IgG IPs. For each IP sample group, the two highest SAINT scores were averaged. Specific PPIs were assigned using an average score of 0.8.

Differential HTT interactions were quantified by precursor intensity-based label-free analysis using Minora in Proteome Discoverer (v2.4.0.305), which assigns features and links them to respective PSMs. A consensus analysis was performed that aggregated individual sample results, which controlled global FDR to 1% at the peptide and protein levels and performed retention time recalibration and feature matching between runs. Additionally, data normalization was performed with the bait protein levels (mouse huntingtin, Htt) and interacting protein ratios (Q140/Q20) were calculated using the protein ratio-based method. Protein abundances were calculated by summed peptide abundances. Proteins were retained that had ≥2 quantitative values in all replicates of at least one sample group. An initial consensus analysis was performed containing four sample groups (Q20-2M, Q140-2M, Q20–10M, and Q140–10M) each with three biological replicates. Results were exported to Excel, and for each sample group, the two samples that minimized the coefficient of variance (CV) between replicates were selected for a second consensus analysis using the same parameters as described above. The results were exported to Excel, and statistical significance between sample group abundances was performed by Student’s t-testing. Interacting proteins were considered differential if |log_2_(Q140/Q20)| > 1.0 and *p*-value < 0.05.

For isotope-labeled data (Q20-2M and Q140-2M), quantification was performed in Proteome Discoverer with the Minora node as above, except an isotope-labeled MS^1^-based quantification method was used that specified lysine-containing peptides could be either the light (^12^C) or heavy (^13^C_6_) isotopologue. In the consensus analysis, peak matching was used to assign nonsequenced light- and heavy-labeled peptides for which the cognate heavy and light isotopologues, respectively, were sequenced by MS/MS and had confidently identified PSMs (<1% FDR) and a valid MS^1^ feature as assigned by Minora in the Processing workflow.

## Results

### Development of HTT-OMNI: an Interactive Platform for HTT PPI Exploration

An important hurdle in extracting biologically relevant HTT PPI targets remains the integration and visualization of new and existing PPI and “omics” datasets. To address this issue, we have developed a deployable, user-friendly web-based application that can simultaneously integrate and visualize information contained within the HINT PPI database and a curated set of HD-relevant omics datasets. We have named this tool the HTT-OMNI (HTT OMics and Network Integration) viewer. An essential aspect of this endeavor has been creating a user-friendly interface that can distill the vast amount of available multi-omics data down to a manageable and visualizable level. To accomplish this, we have taken advantage of the HoloViz Python metapackage, which provides powerful tools for browser-based data visualization and application dashboarding. Specifically, we leveraged the Panel library (27) to develop the application components and the HoloViews library (28) for interactive network and omics data plotting. To highlight the main functionalities of HTT-OMNI, we provide a video tutorial that guides users through its different features and capabilities (supplemental Movie S1).

**Fig. 1:**
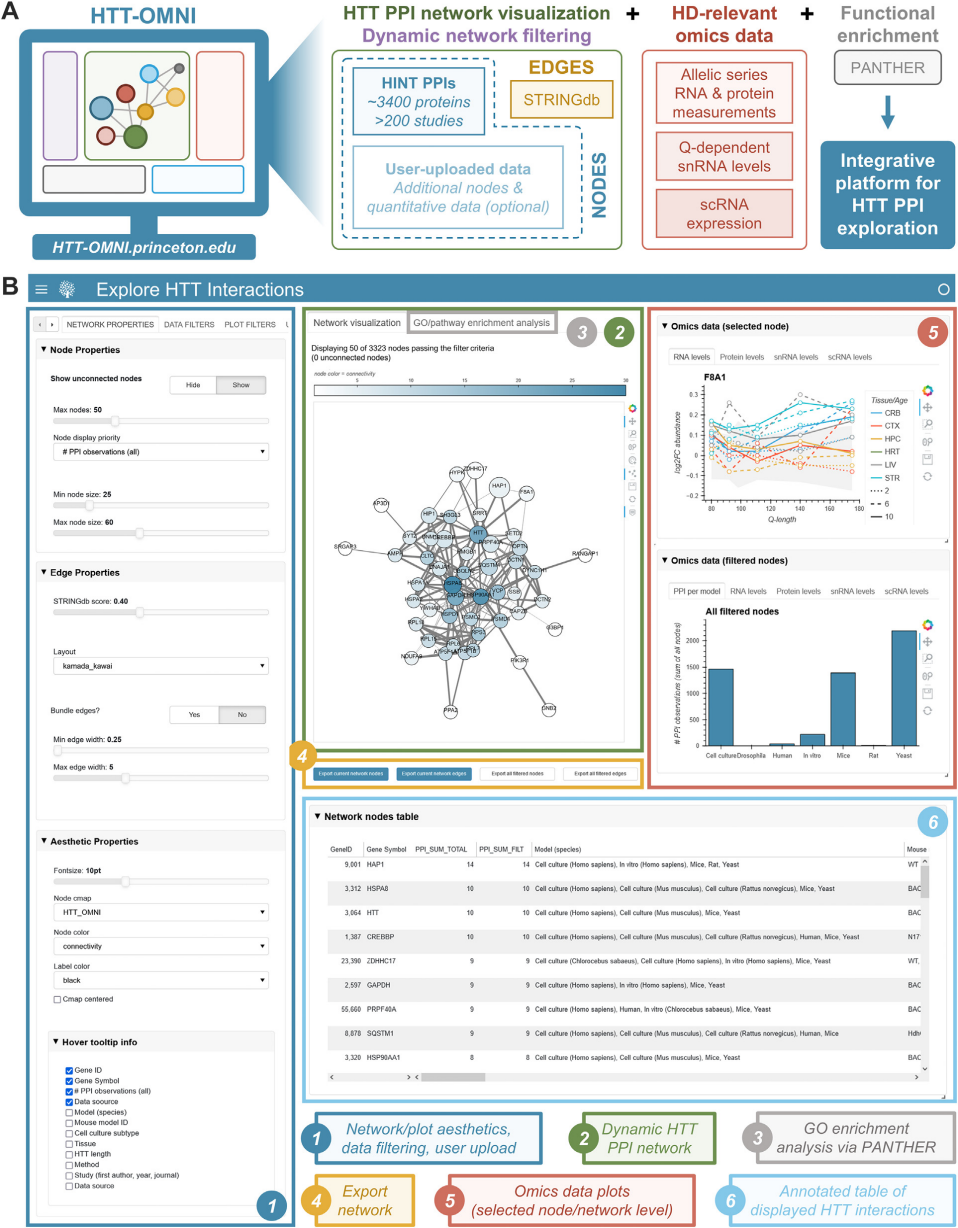
**HTT-OMNI is an interactive platform for HTT PPI and omics exploration.***A*, schematic overview of the HTT-OMNI platform. *B*, annotated screenshots highlighting different features of the main HTT-OMNI interface.

The main elements of HTT-OMNI consist of a functional network of HTT PPIs and linked panels for visualizing HIP-associated HD omics resources. Currently, an extensive repository of >9000 reports of HIPs from 239 studies is available through the HDinHD portal (7), and independently, several large-scale omics studies in mice provide systems-level perspective on disease pathogenesis (16, 17, 18). Yet, for HTT-OMNI to leverage these rich resources, we needed to generate a unified “HINTomics” database. To build this resource, we first obtained the most recent HINT database and supplemented it with recently published PPI studies (12, 13) (supplemental Table S1). Next, we generated an HDomics database from bulk proteome and transcriptome measurements from the mouse allelic series (AS) HD model (16) in combination with polyQ-dependent single-nucleus RNA (snRNA) measurements from the striatum of an HTT^Q175^ HD mouse model (17) and single-cell RNA measurements from the striatum of wild-type mice (18) (supplemental Table S2). These independent omics datasets represent a valuable resource to the HD community as the mouse models are all similarly constructed with knock-in of control or disease-causing CAG expanded alleles of huntingtin, which results in a progressive disorder in mice that recapitulates many aspects of the transition from presymptomatic to manifest HD in the human disease (29, 30). In both the supplemented HINT dataset and the HDomics database, we mapped nonhuman targets to their human homolog (if known) and generated the HINTomics database by annotating the HDomics database with positive PPIs in HINT. Using this unified resource, a protein detected in at least one PPI study in the supplemented HINT dataset is available as a node in the HTT-OMNI network viewer, and edges between the nodes represent known interactions that have been imported from the STRINGdb (22) database (Fig. 1, *A* and *B*).

**Fig. 2:**
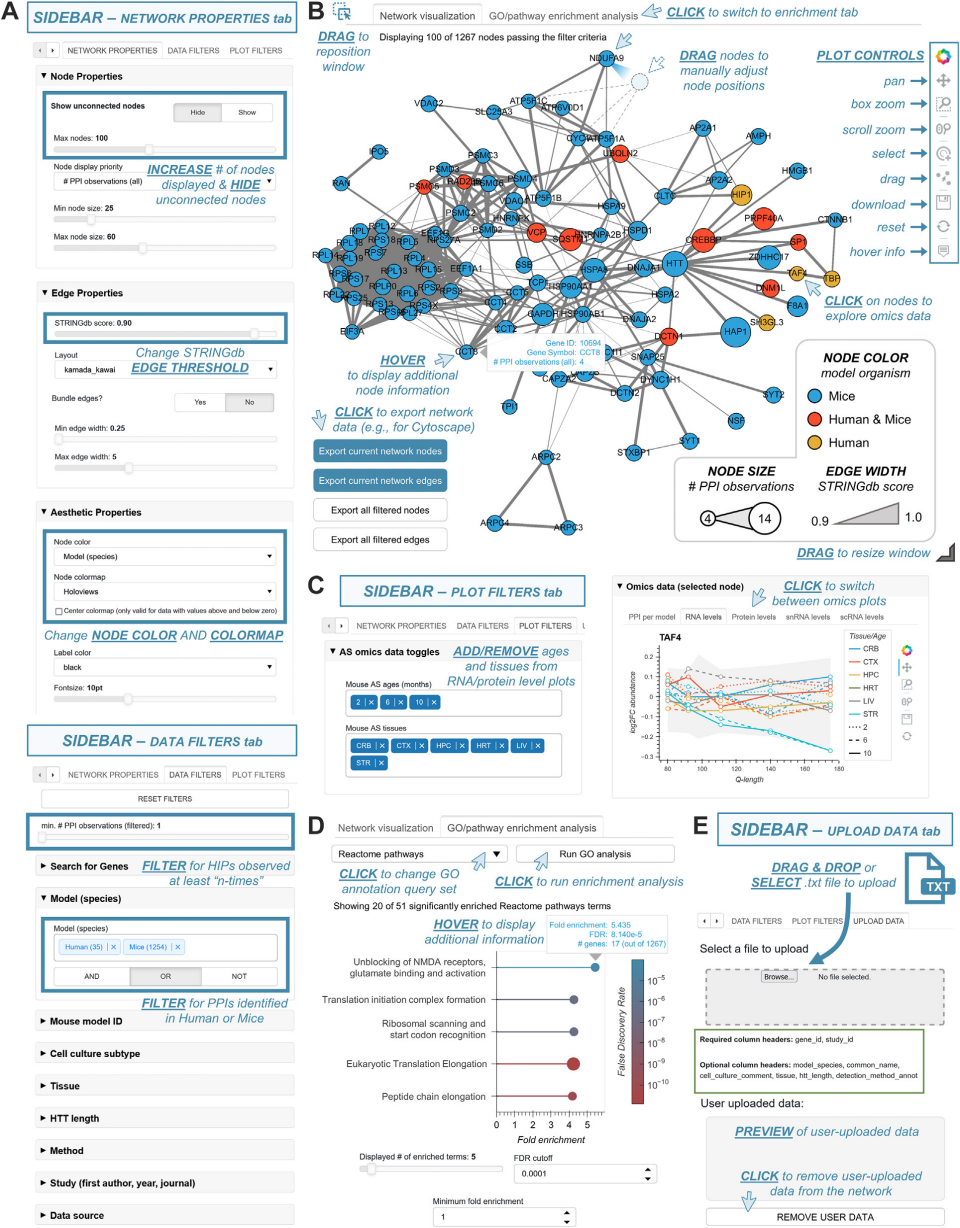
**The HTT-OMNI interface is intuitive and user-friendly.***A*, HTT-OMNI sidebar functionalities that include configuration of network aesthetics (*top*) and filtering of interactions by experimental metadata (*bottom*). *B*, HTT-OMNI network view after applying the settings from panel *A*. Various hover, click, drag, and plot control functionalities are annotated across the network. *C*, HTT-OMNI provides control over which mouse tissues and ages are visible in the RNA and protein levels “omics” graphs. *D*, gene ontology (GO)/pathway enrichment analysis by HTT-OMNI. Available annotation query sets include full and slim versions of GO biological process, GO molecular function, and GO cellular component, as well as Panther and Reactome pathways. *E*, HTT-OMNI provides the ability for users to upload their own datasets. Simply drag-and-drop or browse to select and upload an appropriately formatted tab-separated file.

A benefit of leveraging the HINT database is that it contains detailed experimental metadata for all PPI observations. Given the value of being able to include or exclude HIPs identified under certain experimental conditions, we designed HTT-OMNI to be able to dynamically filter functional networks in real time across different model systems, HTT construct lengths, disease models, tissues, experimental approaches, and studies (Fig. 2*A*). HTT-OMNI also provides the ability to only show HIPs that have been identified in at least a certain number of studies (Fig. 2*A*). Furthermore, users can also configure different aesthetic network properties, such as how many nodes are visible, how nodes are colored and their corresponding colormap, a confidence threshold for edges imported from STRINGdb, edge bundling, and the graph layout algorithm (Fig. 2*A*). We also provide not only the capability to export the network as a scalable vector graphic image but also the ability to download the network nodes and edges as text files that can be externally uploaded to other network visualization software (*e.g.*, Cytoscape (31)) (Fig. 2*B*). Ultimately, the PPI network rendered by HTT-OMNI provides a dynamic interface for user interaction with HTT PPIs, with click, drag, and hover functionalities that augment the user experience (Fig. 2*B*).

In addition to the HTT interaction network described above, our application also provides intuitive, reactive access to the HD-relevant omics datasets within HINTomics (see above) (Fig. 2*C*). Access to measurements within these datasets for an individual PPI is facilitated by clicking on a node within the interaction network, which then renders the corresponding data in the “Omics data (selected node)” panel (Fig. 2*C*). Furthermore, we provide access to this data at a network-aggregated level by computing the sum of all measurements or median measurement (depending on the data type) across all nodes in the filtered network view, which can be viewed in the “Omics data (filtered nodes)” panel. To assist with interpretation of functional gene clusters within a given filtered network view, we also provide direct access to gene ontology (GO) and pathway overrepresentation analysis *via* RESTful API querying of the PANTHER (23) database (Fig. 2*D*).

In addition to filtering and exploring existing HIPs, another key function of our application is to aid in the interpretation and analysis of newly generated HTT PPI datasets that have not yet been integrated into the HINT database. We have therefore included functionality to allow users to upload their own datasets into the application, which allows for the visualization of overlap between newly generated datasets and existing HINT PPIs (Fig. 2*E*). By including “QUANT_” as a tag at the beginning of any column header (*e.g.*, “QUANT_abundance”), users can optionally associate custom quantitative (*e.g.*, fold change, abundance, etc.) or categorical annotations for genes/proteins within their datasets. These user-defined annotations are then added to the “node color” dropdown options (see Fig. 2*A*) and can be visualized as overlays on top of the PPI network. Given that the consolidated omics data (transcriptome, proteome, snRNA-seq, single-cell RNA sequencing) contain measurements for over 15,000 targets (see Experimental Procedures and supplemental Table S2), users can explore omics data for their uploaded data, even when a given gene was not initially present in the base HTT-OMNI network (*i.e.*, HINT database containing ∼3400 HIPs). Altogether, HTT-OMNI brings together HTT PPIs and HD-relevant omics data into a unified platform for data exploration, analysis, and visualization. In the following sections, we highlight specific use cases that demonstrate the functionalities of HTT-OMNI for (1) filtering existing HTT PPIs based on experimental metadata, (2) visualizing the relationships between HIPs and genetic disease modifiers and their multiomic landscape, and (3) analyzing a user-generated dataset of HTT PPIs from mouse cortex samples that has not been previously reported and is not present in HINT.

### Filtering Existing HTT PPIs Based on Experimental Metadata and Visualization of Omics Patterns

**Fig. 3:**
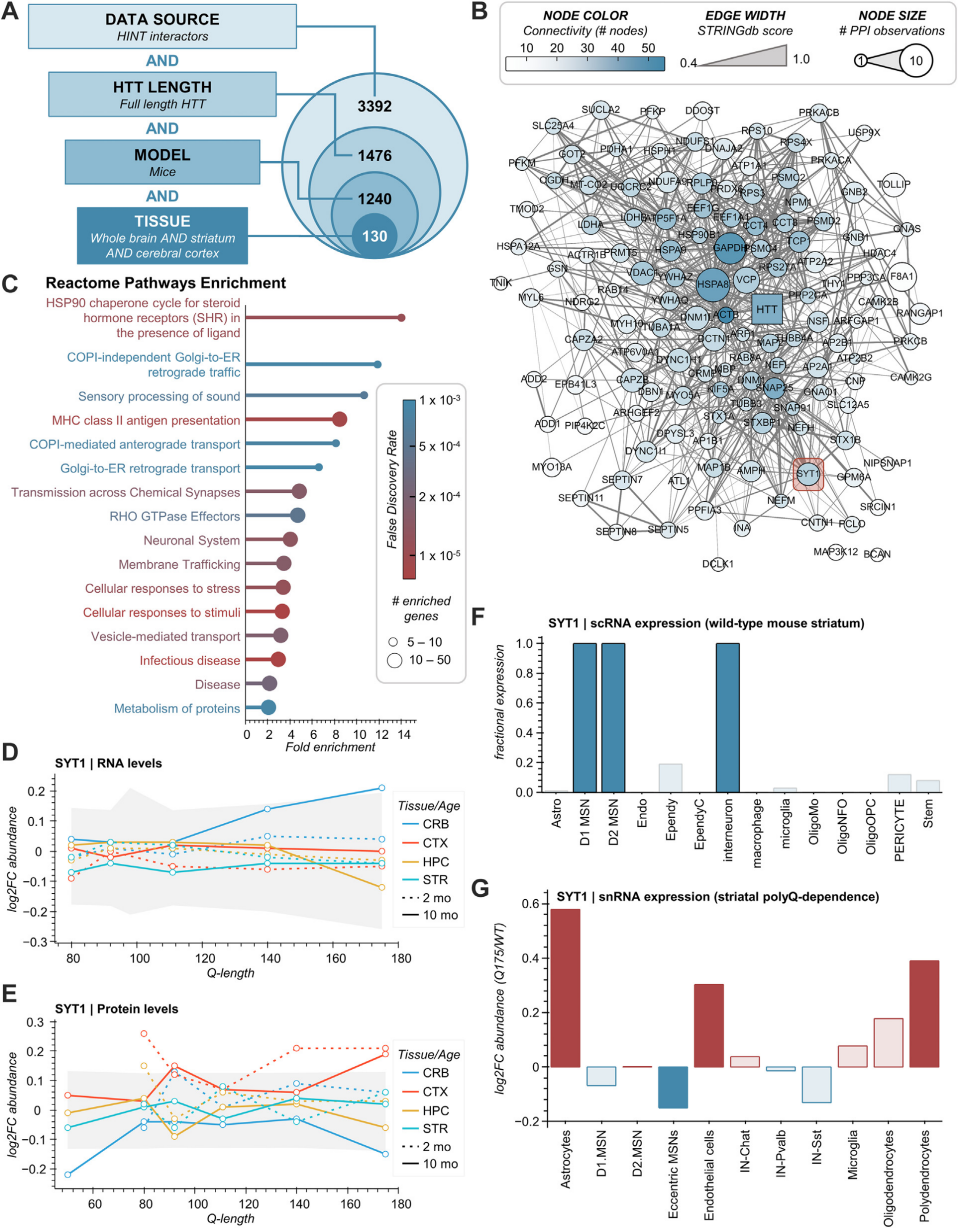
**Filtering existing HTT PPIs based on experimental metadata and visualization of omics patterns.***A*, concentric Venn diagram depicting the number of HTT interactors that pass the following filters: detected as an interactor with full-length HTT, in mice models, and in the whole brain, striatum, and cerebral cortex mouse tissues. *B*, the resulting HTT interaction network after applying the filters described in panel *A*. *C*, reactome pathway overrepresentation analysis of the filtered interaction network depicted in panel *B*. *D*–*E*, SYT1 RNA (*D*) and protein (*E*) levels across different mouse tissues, ages, and Q-lengths. *Gray-shaded boxes* represent a *bounding box* for 90% of the underlying data (*i.e.*, if a datapoint is outside of these bounds, it is in the *upper* or *lower* 5% of the data at that Q-length). *F*, SYT1 scRNA expression levels across different striatal cell subtypes in wild-type (WT) mouse striatum. *Dark blue bars* indicate ≥80% (value ≥0.8) of the analyzed single cells expressed SYT1. *G*, SYT1 snRNA expression levels for Q175 vs. WT mouse models across different striatal cell subtypes. *Dark blue* or *dark red bars* indicate cell types with a |log_2_(Q175/WT)| ≥ 0.15 (>10% increase or decrease for Q175 relative to WT). CRB, cerebellum, CTX, cortex, HPC, hippocampus, STR, striatum; scRNA, single-cell RNA; snRNA, single-nucleus RNA.

Having described the development of HTT-OMNI, we next wanted to demonstrate its utility within the context of analyzing existing HTT PPI studies. Given that such studies have been conducted within a wide variety of experimental contexts, the ability to filter/visualize potential HTT PPIs based on this associated metadata is of high value. Not only does HTT-OMNI facilitate the specific inclusion or exclusion of certain experimental criteria, but it also allows for this metadata to be simultaneously visualized as a user-selected color mapping for nodes in the PPI network (Fig. 2*B*). For example, HTT PPIs can be filtered for those identified only with full-length HTT, in mice models, and in whole brain AND striatum AND cerebral cortex mouse tissues. Applying these filter settings within HTT-OMNI yields a refined set of 130 HTT-interacting proteins (out of ∼3400 proteins in HINT) that have been identified as HTT interactions between 1 and 10 times across all HINT studies (Fig. 3, *A* and *B*). This subset of HIPs formed a highly interconnected network of 128 nodes (STRINGdb interaction scores ≥0.4), with only two HIPs (BCAN and MAP3K12) being unconnected. As expected, given its large number of known interactors, HTT exhibited many connections within this network, including a connection to the well-known HIP F8A1 (32, 33) (also known as HAP40). Within this filtered network, a small subset of these HIPs display high connectivity with other HIPs, suggesting that these targets could serve as hubs of HTT interactions (Fig. 3*B*, *node color*). For example, HSPA8, GAPDH, and SNAP25 are each connected to more than 35 other nodes within this subnetwork. The functionality of HSPA8 and GAPDH in modulating the cellular toxicity of expanded polyQ HTT has been well studied (34, 35), and SNAP25 has been investigated in the context of neurodegenerative diseases (36). Taken together with their numerous physical and functional connections to other HIPs and across the proteome underscores one of the challenges in considering “well connected” HIPs as targets for therapeutic intervention.

For larger interaction networks, *e.g.* >100 nodes, summarizing the functionality at the pathway level is often informative. Using the GO/pathway enrichment analysis tool in HTT-OMNI, 16 Reactome Pathway terms were found enriched (*p* ≤ 0.001), many of which are associated with cellular vesicle trafficking and synaptic transmission (Fig. 3*C*). These pathways are consistent with known cellular functions of HTT (3), underscoring the value of filtering HIPs identified from relevant tissues of HD mouse models.

One of HTT-OMNI’s features is to efficiently link and visualize omics relationships at the network and individual HIP levels. At the network level, we observed that, on average, these HIPs did not show strong correlations with polyQ-length at the respective transcriptome and proteome levels (supplemental Fig. S1, *A* and *B*). Consistent with this, striatal snRNA levels in Q175 vs WT mice were not dramatically altered across most striatal cell subtypes (supplemental Fig. S1*C*). However, the genes corresponding to these PPIs were found to be preferentially expressed in D1 and D2 medium spiny neurons (MSNs), as well as interneurons from the striatum of WT mice (supplemental Fig. S1*D*). An example of omics changes at the individual protein level was observed for the protein synaptotagmin-1 (SYT1), an essential mediator of fast synaptic neurotransmitter release and endocytosis. Bulk mRNA and protein levels for SYT1 in the allelic series HD mouse models show subtle polyQ-dependent effects, with RNA levels increasing with expanded polyQ in the cerebellum at 10 months of age (Fig. 3*D*) and protein levels potentially showing an increase in the cortex (Fig. 3*E*). At the single-cell level, SYT1 showed a strongly accentuated preference for D1 and D2 MSNs and interneurons compared to the whole network (Fig. 3*F* vs supplemental Fig. S1*D*). Although astrocytes have lower relative levels of SYT1 whole cell expression than other cell types in the striatum, this cell type showed the largest relative polyQ-dependent increase in snRNA expression levels (Fig. 3*G*).

Our comparison of omics relationships for HIPs at the network and individual levels raises an interesting question of how often they are targets of regulation at the RNA or protein level by expanded polyQ HTT. To explore this question, we used the “Search for Genes” function of HTT-OMNI to refine the mouse brain HIP network (*e.g.*, Fig. 3*B*) by the genes/proteins that were differential in both the transcriptomes and proteomes of allelic HD mouse models (16). In total, 788 gene symbols with human homologs were associated with differential targets from the “continuous Q” statistical analysis (see Experimental Procedures and supplemental Table S3). After filtering, only 14 of these targets were shared with the mouse brain HIP network (supplemental Fig. S1*E*). The network level omics dataset for this small subset of HIPs shows on average, a decreasing polyQ-dependent RNA expression in the striatum of older mice (supplemental Fig. S1*F*). Moreover, searching the complete HINT database (3392 nodes) for these 788 differential targets only returned 292 HIPs, showing that less than 10% of HIPs may be coordinately regulated at the proteome and transcriptome levels. Overall, the ability of HTT-OMNI to apply experimental metadata and external gene list filters and visualize network and omics datasets supports a hypothesis that proteins within the HTT interactome are rarely targets of differential expression/abundance at the transcriptome and proteome levels.

### Network and Multiomics Interrogation of HTT PPIs Through the Lens of Genetic Disease Modifiers

**Fig. 4:**
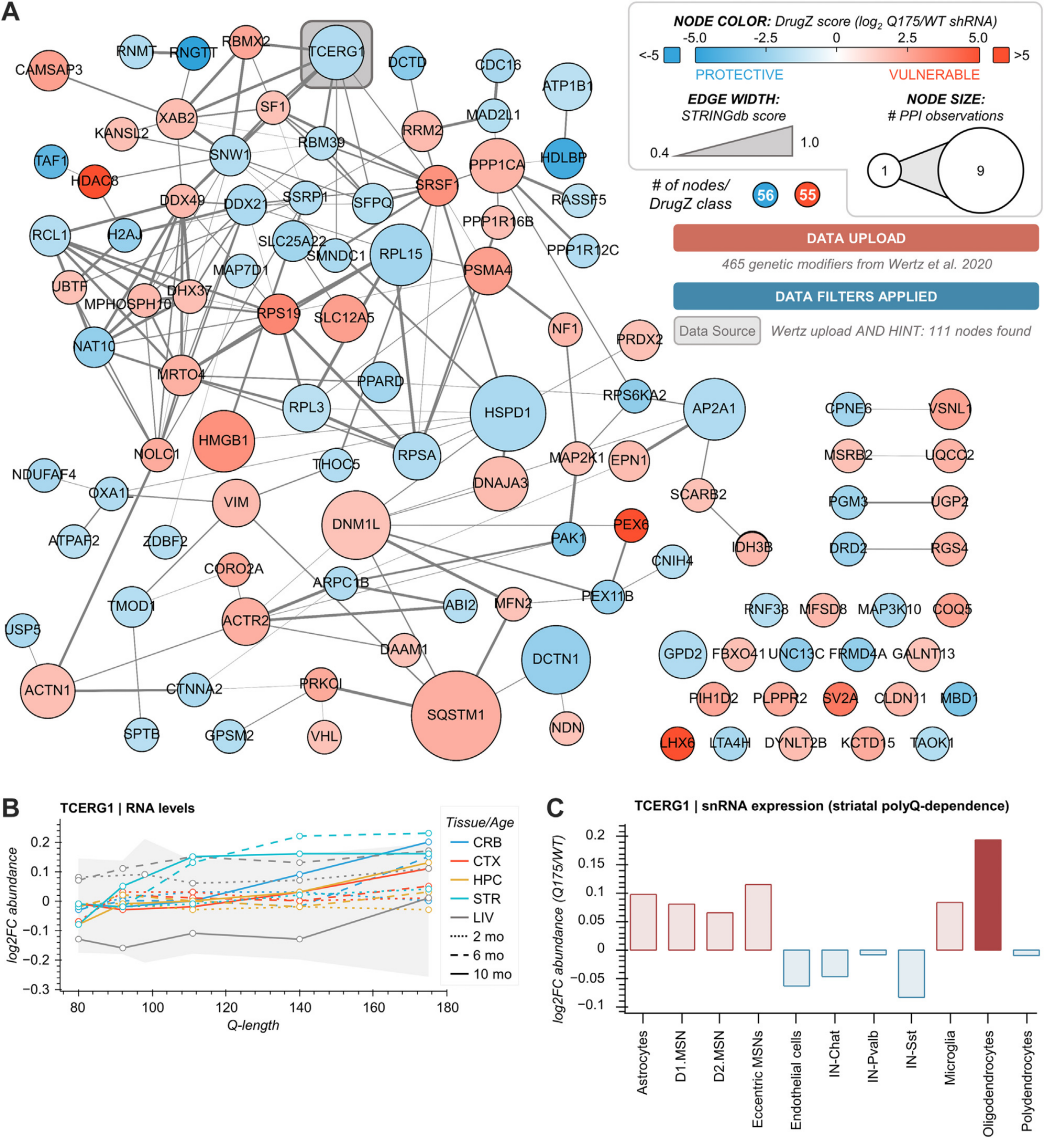
**Network and multiomics interrogation of HTT PPIs through the lens of genetic disease modifiers.***A*, HTT-OMNI network of 111 genetic modifiers from Wertz *et al.* 2020 that overlap with HTT interactors in the HINT database. Node color indicates the relative DrugZ score for a given target, a measure of the gene’s potential protective (negative value) or vulnerable (positive value) modification of mHTT-induced cell toxicity. *B*, TCERG1 RNA levels across different mouse tissues, ages, and Q-lengths. *Gray-shaded boxes* represent a bounding box for 90% of the underlying data (*i.e.*, if a datapoint is outside of these bounds, it is in the *upper* or *lower* 5% of the data at that Q-length). *C*, TCERG1 snRNA expression levels for Q175 vs. WT mouse models across different striatal cell subtypes. *Dark blue* or *dark red bars* indicate cell types with a log_2_ fold change value ≥0.15 (>10% increase or decrease for Q175 relative to WT). CRB, cerebellum; CTX, cortex; HPC, hippocampus; LIV, liver; STR, striatum; snRNA, single-nucleus RNA.

While the previous use case suggested that HIPs are not often regulated by polyQ length in terms of their RNA expression or protein abundance, this does not imply that HIPs are not relevant to disease progression. In studies from our laboratory and others, the genes of HIPs have been shown to be modifiers of disease progression in HD animal models (12, 37, 38). To visualize and interrogate the HIPs that are potential genetic modifiers of HD, we leveraged a recent study from Wertz et al (19), which identified disease-relevant genetic modifiers using genome-wide approaches in mouse models of HD. For this use case, we used HTT-OMNI to display HIPs that were common to a list of candidate HD modifiers identified from the zQ175 full-length m*HTT* knock-in mouse model (19), herein termed “PPI genetic modifiers” (supplemental Table S4). In total, 111 HIPs could be classified as PPI genetic modifiers and were assembled into a functional interaction network that was overlaid with each candidate’s respective DrugZ score, a measure of the gene’s potential protective (negative value) or vulnerable (positive value) modification of mHTT-induced cell toxicity (Fig. 4*A*). This representation illustrated that essentially equal numbers of PPI genetic modifiers were classified as protective *versus* vulnerable, and these proteins included frequently reported HTT interacting proteins in HINT (Fig. 4*A*, node size) across several model systems (supplemental Fig. S2*A*), such as sequestosome 1 (SQSTM1), the mitochondrial chaperone HSPD1, dynamin-1-like protein (DNM1L/DRP1), and Transcription elongation regulator 1 (TCERG1). A majority of these PPI genetic modifiers (n = 66) had at least one subcellular localization annotation as the nucleus (supplemental Figs. S2*B* and S3), as documented by UniProt (20) and/or the Human Protein Atlas (21). Among the PPI genetic modifiers that have been frequently observed and nucleus annotated, TCERG1 stood out as a notable target (highlighted in Fig. 4*A*). TCERG1 contains a Gln-Ala repeat track and can modulate transcription elongation and mRNA splicing through interaction with RNA Pol II and splicing factor SF1 (39). The HTT–TCERG1 interaction has been documented in yeast two-hybrid and *in vitro* studies (supplemental Fig. S4*A*) (40, 41, 42, 43). At the omics level, polyQ-dependent regulation of TCERG1 is apparent in the allelic series mouse models at the RNA level (Fig. 4*B*). Specifically, the striatum showed the greatest relative increase, though most tissues show a positive correlation with polyQ-length, particularly at 10 months of age (Fig. 4*B*). PolyQ-dependent snRNA expression of TCERG1 suggests this striatal expression increase is driven primarily by oligodendrocytes, with a smaller contribution by astrocytes and MSNs (Fig. 4*C*). However, these data also indicate opposing lower magnitude decreases in TCERG1 RNA across interneuron cell types. This could be biologically relevant as under homeostatic conditions, TCERG1 expression has preferential expression in interneurons, closely followed by MSNs (supplemental Fig. S4*B*). In contrast to RNA levels, striatal proteome levels of TCERG1 in the allelic series models are negatively correlated with polyQ length at 6 and 10 months of age (supplemental Fig. S4*C*). In the context of HD patients, the repeat track of TCERG1 was initially found to have a slight contribution to disease age of onset (40), while more recent genome-wide studies by GEM-HD found that TCERG1 was one of the most significant age-of-onset hits (44). Overall, the ability of HTT-OMNI to allow filtering of HINT by non-PPI datasets, exemplified here by HD genetic modifiers, and seamless access to multiple omics-level studies, allowed us to perform a targeted extraction of systems-level dynamics of TCERG1.

### Functional Network Visualization of Tissue- and polyQ-Dependent HTT PPIs From the Cortex and Striatum

Having demonstrated the utility of HTT-OMNI for analyzing existing PPI datasets, we next aimed to show how HTT-OMNI can analyze a user-supplied quantitative IP-MS dataset that has not been previously reported. In individuals with HD, impaired neuronal communication is observed between cells in the striatum and cortex tissues, particularly in early stages of the disease (11). Therefore, comparison of dysregulated HIPs in the cortex of HD mice to existing PPI datasets in HD mouse models can provide insights into cellular functions or pathways that may be common or tissue specific. To this end, we immunoaffinity purified HTT from mouse cortex expressing normal (3xFLAG-HTT Q20) or mutant HTT (3xFLAG-HTT Q140) at 2 and 10 months of age. We have selected these two ages as molecular and phenotypic analysis of HD mouse models at these time points show a progressive disease course (16, 29, 30). For example, in HD mouse models with various polyQ lengths, transcriptional dysregulation is relatively modest at 2 months of age (∼100 RNAs), while at 10 months of age, ∼3000 genes become dysregulated (16). Moreover, early neuron dysfunction in HD mice progresses to neuronal loss at later disease stages (29, 30). Additionally, we have recently demonstrated age-dependent dysregulation of specific HIPs in the striatum of HD mice at these two ages (12).

**Fig. 5:**
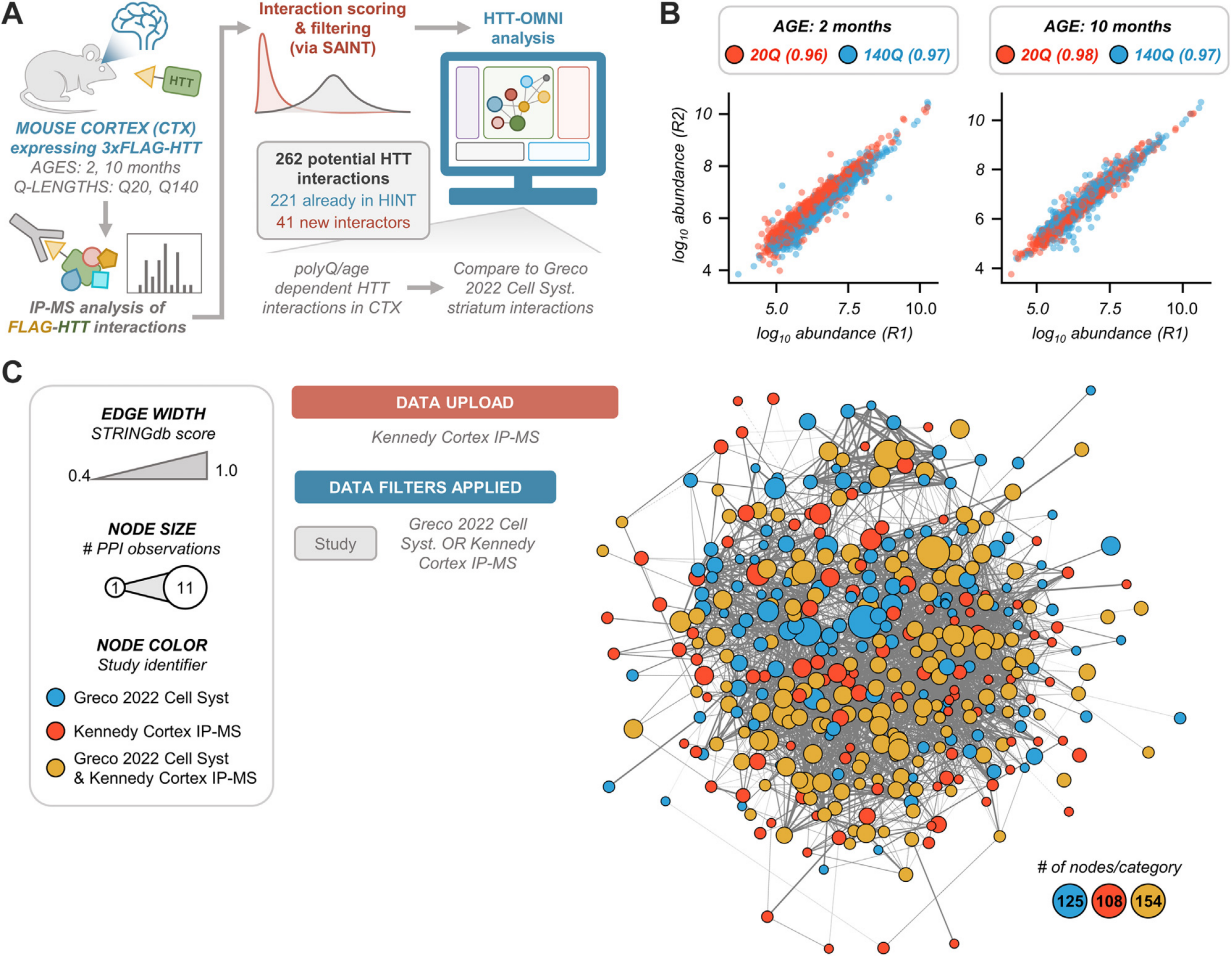
**Characterization of HTT interactions in mouse cortex *via* IP-MS at 2 and 10 months of age.***A*, schematic depiction of HTT IP from mouse cortex samples and subsequent analysis and visualization of specific interactions (as determined by SAINT) *via* HTT-OMNI. *B*, reproducibility of protein abundances from two biological replicates of cortex HTT IPs. The number next to each line in the legend indicates the Pearson correlation for that set of samples. *C*, overlap between cortex (this study) and striatum (Greco *et al.* 2022) IPs, as visualized by HTT-OMNI. Node color indicates whether a given node was identified as a specific HTT interactor in the cortex (Kennedy Cortex IP-MS), striatum (Greco 2022 Cell Syst), or both (Greco 2022 Cell Syst & Kennedy Cortex IP-MS). All HTT-interacting proteins detected at any age or Q-length are depicted in this network.

Following immunoaffinity purification of HTT protein complexes, we relatively quantified the co-isolated proteins using a previously described label-free IP-MS approach (Fig. 5*A*, supplemental Table S5 and S6) (12). Nonspecific interactions were filtered from bait IPs using the SAINT algorithm (26), producing 262 potential HTT interactions across all sample groups (Fig. 5*A*). Interaction abundances were measured by precursor-based label-free quantification (LFQ) and normalized to HTT, resulting in high correlation in protein abundances between replicate IPs (Fig. 5*B*, and Experimental Procedures). As not all user datasets will contain quantitative data, we first demonstrated how to perform a qualitative comparison of PPIs identified between a user’s data and an existing study within HTT-OMNI/HINT. The 262 HIPs were uploaded with their human gene symbols to HTT OMNI and an annotation column that links each HIP to a “study_id” of “Kennedy Cortex IP-MS.” HTT-OMNI indicated that 221 HIPs have been observed by at least one other study, while 41 represent previously unreported HTT PPIs. Within HINT, a recent study from Sap et al (13) used cross-linking IP-MS to identify 57 HIPs from the cortex of HD mice. Visualization of these two datasets in HTT-OMNI (supplemental Fig. S5*A*) showed a common set of 10 HIPs (supplemental Fig. S5, *B* and *C*). Given the different isolation conditions for XL-IP-MS (*e.g.*, lysis buffer and workflow), these shared interactions may represent direct interactions, which is supported by the higher proportion of overlap (81%) of “Kennedy Cortex IP-MS” with HINT compared to Sap *et al.* (supplemental Fig. S5, *B* and *C*).

Next, to gain insights into possible tissue-dependent HIPs, we compared this cortex dataset to our recently reported HIP dataset from the striatum (279 HIPs), which we annotated in HTT-OMNI with the study identifier “Greco 2022 Cell Syst”. Application of an “OR” “Study” filter between the two datasets produced a unified interaction network of 387 HIPs (Fig. 5*C*). At the network level, node color was mapped by the different study identifiers to visualize the 154 shared HIPs *versus* the 125 and 108 HIPs that were scored as HIPs specifically enriched within the striatal or cortex datasets, respectively (Fig. 5*C*). Given that both the cortex and striatal datasets have associated LFQ interaction abundances, we were able to directly compare the impact of expanded polyQ and age on changes in interaction levels. We present two complementary strategies to visualize differential interactions when performing pairwise comparisons of different experimental conditions.

**Fig. 6:**
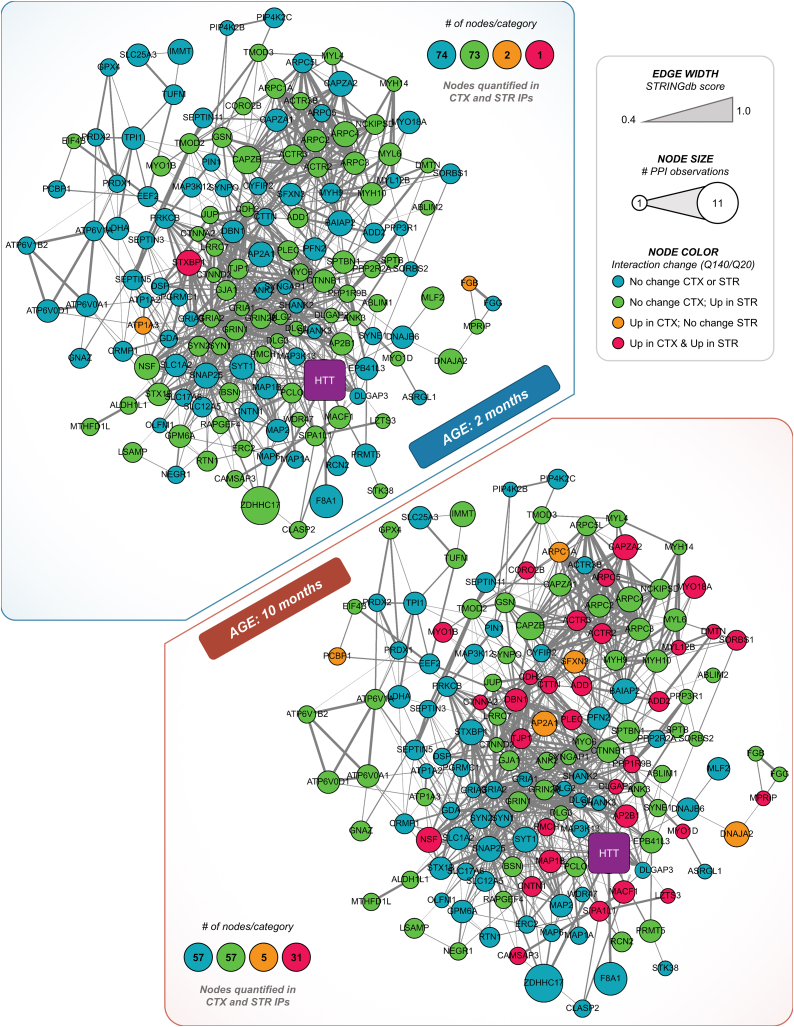
**Functional network visualization of polyQ-dependent interactions shared between cortex and striatum.** HTT interactors that are specific (SAINT score ≥0.8) in both striatum (STR) and cortex (CTX), classified by polyQ dependence at 2 or 10 months. Node color indicates whether the interaction level was unchanged or increased (Up) in the cortex or striatum at a given age. PPIs were considered “Up” if their respective log2 fold change (Q140/Q20) was ≥1 and they had a *p*-value < 0.05 as determined by two-tailed Student’s *t* test.

In the first approach, we focused on the common interactions that were quantified in both the striatum and cortex (Fig. 5*C*, *orange nodes*). We performed categorical assignment of differential polyQ (Q140/Q20) interaction abundance based on fold-change and *p*-value thresholds of ≥ ±2.0 and <0.05, respectively (supplemental Table S7). HIPs were then visualized as described above, separately visualizing the polyQ abundance classifications for 2 and 10 months (Fig. 6). These network visualizations reveal several key points regarding polyQ-dependent interactions: (1) increases in interactions predominate in the common pool of cortex and striatum HIPs, (2) at the same age, there are more differential interactions in the striatum and few uniquely differential interactions in the cortex, and (3) the number of differential cortex and striatum interactions increases with age. As striatal neuronal dysfunction is an early hallmark of the disease, we used the “Search for Genes” Data filter to visualize the 2-month subnetwork of striatum-specific differential PPIs that could contribute to early disease pathogenesis (supplemental Fig. S6*A*). The PPIs underlying these functions are largely represented by two densely connected modules containing subunits of the Arp2/3 complex (ACTR3B, ARPC1A, ARPC2, ARPC3, ARPC4), glutamate receptors (GRIN1, GRIN2B, GRIA1, GRIA2), catenins (CTNNB1, CTNND2), and regulators of vesicle fusion and recycling (*e.g.*, STX1B, SYN1, SYN2, and PCLO).

**Fig. 7:**
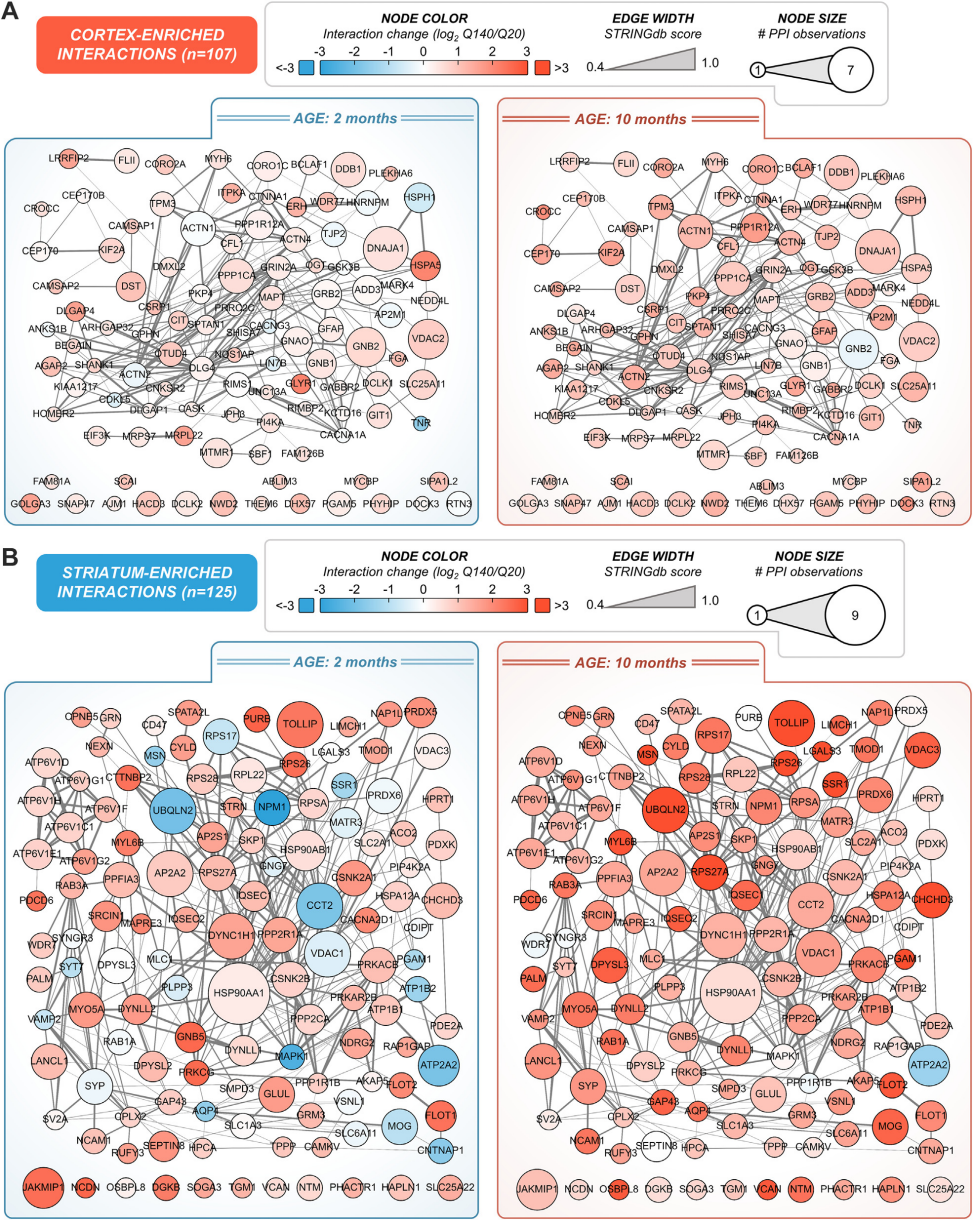
**Visualization of polyQ-dependent interactions that are preferentially enriched in cortex or striatum.***A*, cortex-enriched HTT PPIs passing a SAINT specificity threshold of at least 0.8 in cortex IP samples, but not in striatum IP samples. Node color indicates the relative change in HTT interaction with a given protein in Q140 vs Q20 mice models. *B*, striatum-enriched HTT PPIs passing a SAINT specificity threshold of at least 0.8 in striatum IP samples, but not in cortex IP samples. Node color indicates the relative change in HTT interaction with a given protein in Q140 vs Q20 mice models.

The second approach we present for network depiction of differential interactions is to directly overlay relative interaction abundances (*i.e.*, log_2_ Q140/Q20 abundances). HIP networks were constructed that contained SAINT-specific PPIs unique to the cortex (Fig. 7*A* and supplemental Table S8) or striatum (Fig. 7*B* and supplemental Table S9) at 2 and 10 months of age (Fig. 7*B*, *left* vs *right*). Interaction fold change was node color coded on the same scale, illustrating the larger magnitude of change observed for putative striatum-specific interactions compared to the more subtle changes in cortical PPIs, which is consistent with our comparison of shared interactions between the cortex and striatum (Fig. 6). Within the cortex networks, several HIPs that have been frequently observed in previous studies were present, including voltage-dependent anion-selective channel protein 2 (VDAC2), guanine nucleotide-binding protein G(I)/G(S)/G(T) subunit beta-2 (GNB2), and serine/threonine-protein phosphatase PP1-alpha catalytic subunit (PPP1CA). Our IP-MS dataset also contained many PPIs that have not been previously observed, including two voltage-dependent calcium channels (CACNA1A and CACNG3) and calmodulin-regulated spectrin-associated proteins (CAMSAP1 and CAMSAP2).

### Defining the Relative Stability of Tissue-Dependent HTT PPIs From the Cortex and Striatum

**Fig. 8:**
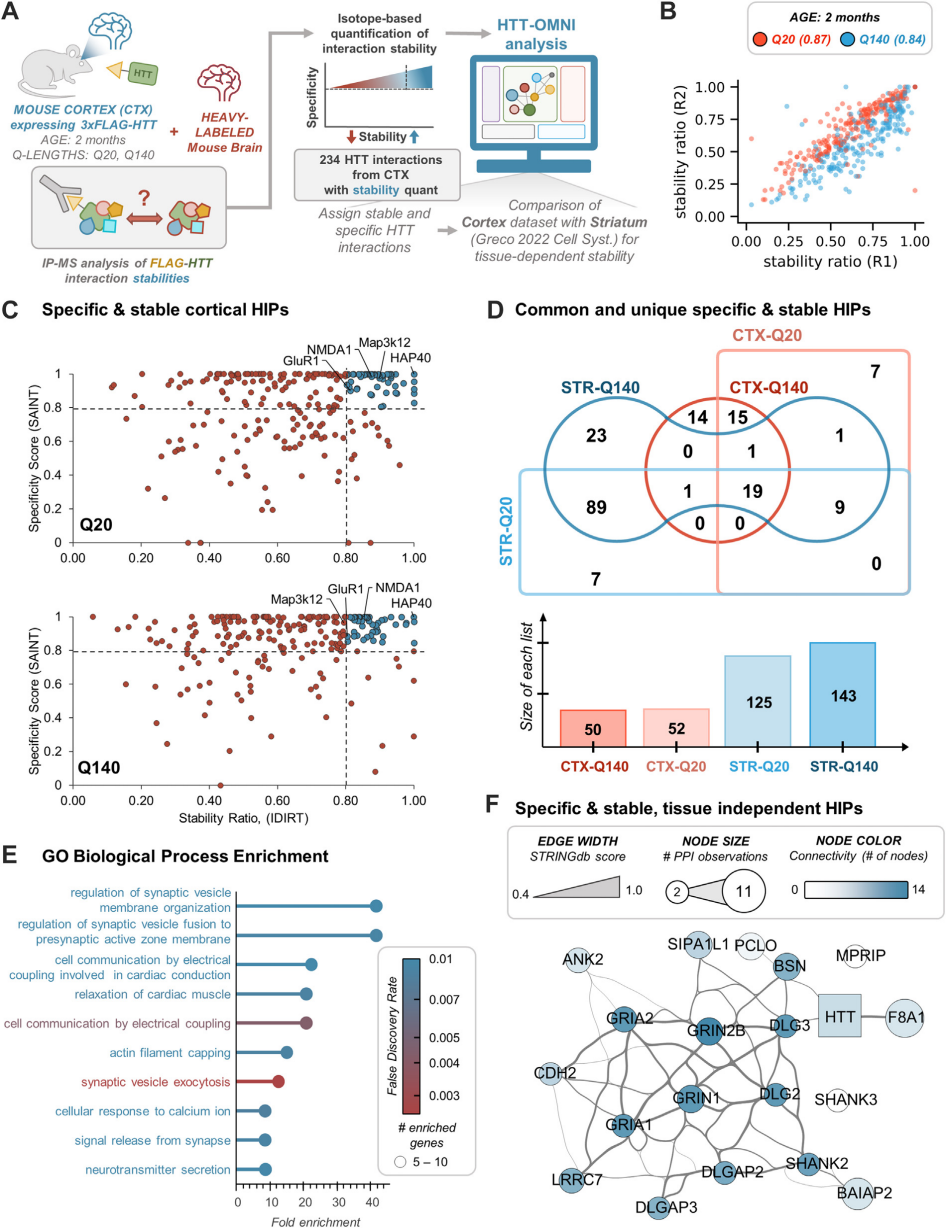
**Visualization and functional analysis of tissue-dependent PPI stability networks.***A*, schematic depiction of isotope-labeled HTT IP from mouse cortex samples mixed with heavy-labeled whole brain. Mass spectrometry analysis was used to determine light/[light+heavy] stability ratios and were compared to SAINT-specific interactions (see Fig. 5) to assign specific and stable HIPs. Analysis and visualization of HIPs was performed by HTT-OMNI. *B*, reproducibility of stability ratios between two biological replicates of isotope labeled HTT IPs. The number in parentheses next to each polyQ-length indicates the Pearson correlation between replicates. *C*, scatterplots of specificity score (SAINT) *versus* stability ratio for quantified HIPs from Q20 and Q140 IPs. HIPs in the *upper-right* quadrant were assigned as specific & stable. *D*, Venn comparison of HIPs assigned specific/stable between tissues and polyQ-lengths. Bar plots show the total number of assigned HIPs per group. *E*, functional overrepresentation of GO biological processes of specific and stable HIPs assigned only to the striatum (n = 23 + 89 + 7 HIPs from D). *F*, HTT-OMNI network of tissue-independent (common) HIPs that were assigned as specific and stable (n = 19 from D).

We have previously shown that the presence of mutant HTT can induce changes in the relative stabilities of HIPs isolated from the striatum (12). To determine whether the presence of alterations in interaction stabilities is a tissue-dependent feature, we aimed to explore the polyQ-dependent stability landscape of HIPs in the cortex. We implemented an approach that merges label-free and metabolic labeling IP-MS (45, 46) to assess both the relative stabilities and specificities of protein interactions (25). For this, we immunoaffinity purified FLAG-HTT from a mixed brain lysate, in which the cortex from FLAG-Htt HD mice was mixed 1:1 (w:w) with heavy (^13^C_6_-Lys)-labeled whole-brain lysates of wild-type and age-matched mice (Fig. 8*A*). As previously shown by studies performed in either cells or mouse tissues (12, 46), the relative quantification of co-isolated proteins in their light (endogenous) and heavy (reference) isotope forms allows for the determination of a relative interaction stability ratio. Ratios closer to one indicate little in-solution exchange, and thereby, more stable interactions, while stability ratios closer to 0.5 represent fast-exchanging or transient associations. Based on our prior observations of changes in HIP relative stability for selective functional classes at 2 months of age (12), we selected this age for comparison with the cortex. In these isotope-labeled IP-MS experiments (n = 2 biological replicates), we quantified ∼90% of the specific HIPs (234/262) that were also measured in LFQ experiments (see Figs. 5, 6 and supplemental Table S5). These HIPs showed good correlation of isotope stability ratios across replicates (Fig. 8*B*). Comparison of relative stability ratios to SAINT-based specificity scores showed that ∼20% of HIPs were both stable and specific for HTT Q20 and Q140 (Fig. 8, *C*, *D* and supplemental Table S10). Moreover, very few HIPs exhibited relative stabilities that were polyQ-dependent, except MAP3K12, which had a lower stability ratio with mutant (Q140) HTT (Fig. 8*C*). Comparison of stable and specific HIPs between the cortex and striatum showed that a greater percentage of HIPs are assigned to this class in the striatum (Fig. 8*D*), with 119 stable and specific HIPs occurring in the striatum. Visualization of the connectivity (supplemental Fig. S6*B*) of this subnetwork and GO analysis (Fig. 8*E*) revealed enrichments in terms associated with synaptic vesicle exocytosis, regulation of synaptic vesicle fusion and membrane organization, and actin filament capping. Finally, we visualized the 19 HIPs ( see Fig. 8*D*) that were common between the striatum and cortex (Fig. 8*F*). Most of these HIPs had known functional relationships, centered around glutamate (NDMA1/NDMA2B) and AMPA (GluR1/GluR2) receptors and factors localized to the postsynaptic density. In summary, HTT-OMNI facilitated the rapid comparison of tissue-derived HTT interactomes through its categorical and quantitative network visualization functionalities and helped pinpoint tissue-specific candidate HIPs for functional validation.

## Discussion

The extensive number of potential HTT interactors that have been identified across hundreds of studies using different organisms, experimental approaches, and perturbations has made it challenging to identify high-value targets for therapeutic design. Moreover, the integration of HIPs with their associated multiomic measurements has not been made easily accessible. Here, we describe HTT-OMNI, a web-based application for HIP network analysis, visualization, and omics data exploration. We have demonstrated the utility of HTT-OMNI for a variety of use cases, highlighting its ability to filter and visualize HIPs by their experimental metadata and to integrate user-uploaded datasets into existing HIPs from the HINT database. Given that HINT serves as the foundation of HTT-OMNI, it will be straightforward to continue updating the base HTT-OMNI interaction network following regular updates to the HINT database. Furthermore, the modular development of the application itself makes the inclusion of additional quantitative modules (*e.g.*, additional omics datasets) feasible.

We also exploited the ability of HTT-OMNI to visualize and derive biological insights from diverse HD datasets. Simultaneous visualization of differential HIPs identified from our previous IP-MS study (in the striatum) and an IP-MS dataset generated here (from cortex) revealed that polyQ-dependent regulation of HIP abundances and stabilities were more prominent in the striatum of HD mice at both 2 and 10 months of age. This may reflect the differential impacts of HD pathogenesis on these two tissues and is consistent with the striatum brain region being especially vulnerable to expanded polyQ-associated cell toxicity (10). More broadly, the integration of OMICS resources to PPI networks suggested that the majority of HIPs are not concurrently modulated at the proteome or transcriptome levels, though continued single-cell analyses may reveal additional cell-type-specific regulatory mechanisms. We also demonstrate that the utility of HTT-OMNI is not limited to IP-MS datasets. Given the simple, but flexible, structure of data uploaded to HTT-OMNI, it possible to use this tool to analyze various systems level HD datasets outside the context of interaction studies (as shown by our analysis of the genetic modifiers from Wertz *et al.* 2020). In summary, HTT-OMNI is poised to grow with the HD field, incorporating new studies as they become available and providing a unified platform for HIP network and omics data exploration.

## Data Availability

The mass spectrometry proteomics data have been deposited to the ProteomeXchange Consortium *via* the PRIDE (47) partner repository with the dataset identifier PXD034158. All HTT-OMNI code is available on GitHub at cristealab/HTT-OMNI.

## Supplemental data

This article contains supplemental data.

## Conflict of interest

The authors declare that they have no conflicts of interest with the contents of this article.

## Supplemental Data

Supplemental Figures

TableS1

TableS2

TableS3

TableS4

TableS5

TableS6

TableS7

TableS8

TableS9

TableS10

MovieS1
