# Supplementary material for: HTT-OMNI: A Web-based Platform for Huntingtin Interaction Exploration and Multi-omics Data Integration
Source: Mol Cell Proteomics. 2022 Aug 3;21(10):100275. doi: 10.1016/j.mcpro.2022.100275 (PMC9540350; doi:10.1016/j.mcpro.2022.100275)
Supplement: Supplemental Figures [file mmc1.pdf]

HTT-OMNI: A Web-based Platform for Huntingtin Interaction Exploration and Multi-omics Data Integration

Michelle A. Kennedy*, Todd M. Greco*, Bokai Song, and Ileana M. Cristea†

** These authors contributed equally*

Supplemental figures S1-S6

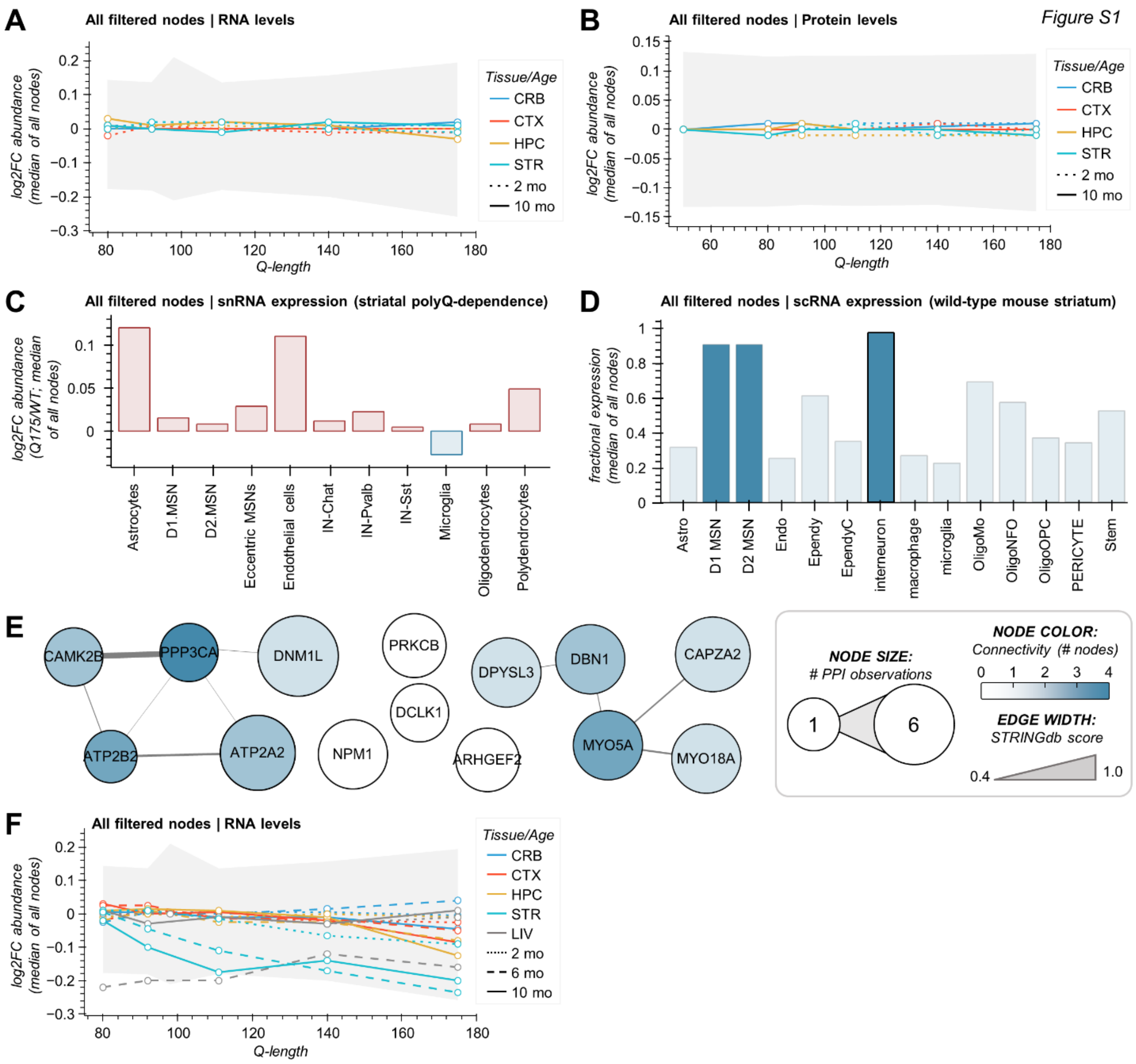

Figure S1: Application of HTT-OMNI for filtering HINT interactions and visualizing HIP-associated omics data, related to Figure 3

A-B) Network-level RNA (A) and protein (B) levels across different mouse tissues, ages, and Q-lengths (median of all nodes in Figure 3B). Grey shaded boxes represent a bounding box for 90% of the underlying data (i.e., if a datapoint is outside of these bounds, it is in the upper or lower 5% of the data at that Q-length); CRB = cerebellum, CTX = cortex, HPC = hippocampus, STR = striatum. C) Network-level snRNA expression levels for Q175 vs. WT mouse models across different striatal cell subtypes (median of all nodes in Fig. 3B). D) Network-level scRNA expression levels across different striatal cell subtypes in wild-type (WT) mouse striatum (median of all nodes in Fig. 3B). Dark blue bars are those with values ≥ 0.8, representing particularly high expression levels. E) Subnetwork of 14 HIPs that were differential in their transcriptomes and proteomes as measured by Langfelder *et al.* 2016. F) Network-level RNA levels across different mouse tissues,

ages, and Q-lengths (median of all nodes in panel E). Grey shaded box represent a bounding box for 90% of the underlying data (i.e., if a datapoint is outside of these bounds, it is in the upper or lower 5% of the data at that Q-length); CRB = cerebellum, CTX = cortex, HPC = hippocampus, STR = striatum, LIV = liver.

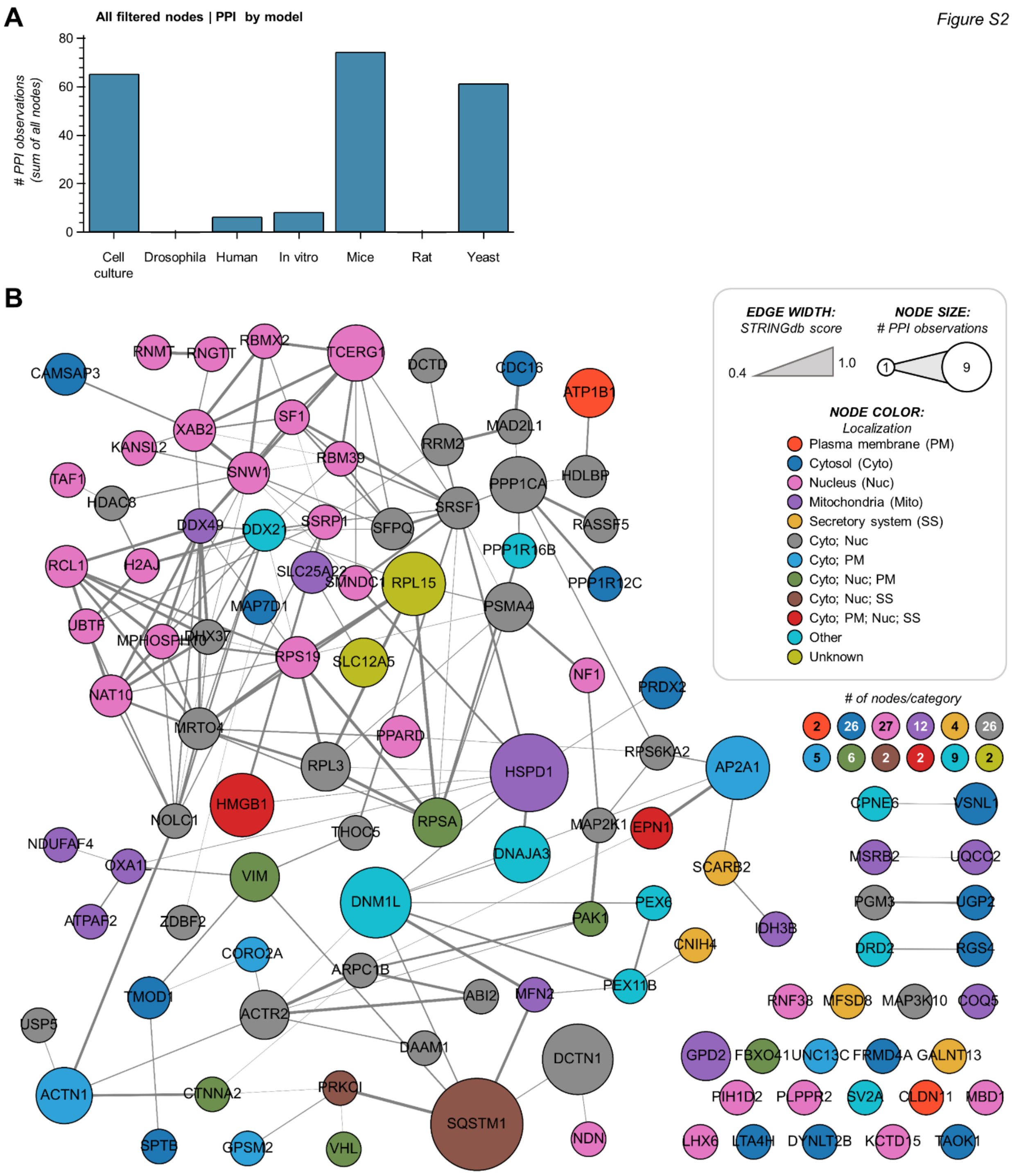

Figure S2: Genetic modifier HIPs PPI observations across models and colored by localization, related to Figure 4

A) Network level PPI observations across different experimental models (sum of all nodes from Fig. 4A). B) HTT-OMNI network of 111 genetic modifiers from Wertz et al. 2020 that overlap with HTT interactors in the HINT database. Node color indicates the target's subcellular localization as annotated by UniProt and the Human Protein Atlas (see Methods).

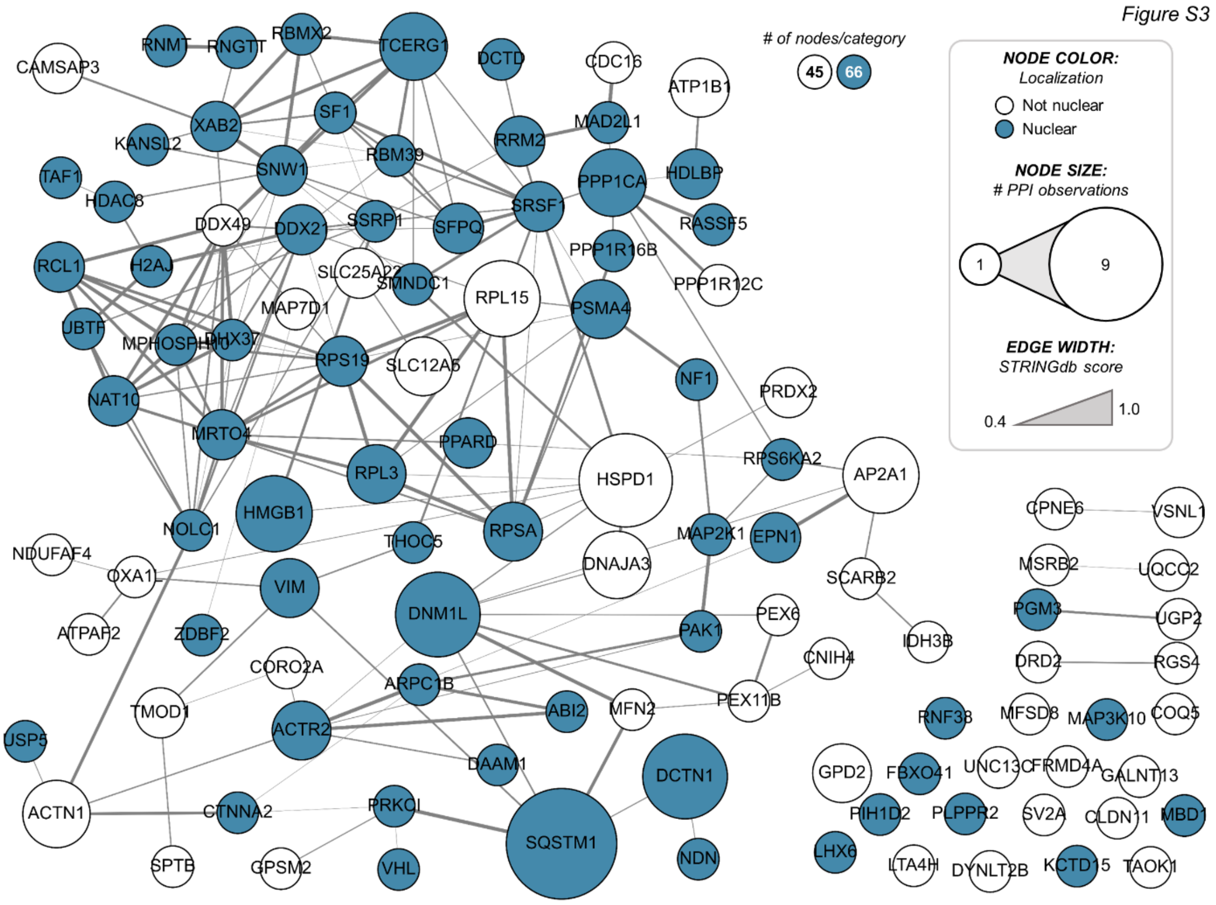

Figure S3: Genetic modifier HIPs—colored by nuclear annotation, related to Figure 4

HTT-OMNI network of 111 genetic modifiers from Wertz et al. 2020 that overlap with HTT interactors in the HINT database. Node color indicates whether a given target has any subcellular localization annotation that is associated with the nucleus. Annotations were sourced from both UniProt and the Human Protein Atlas (see Methods).

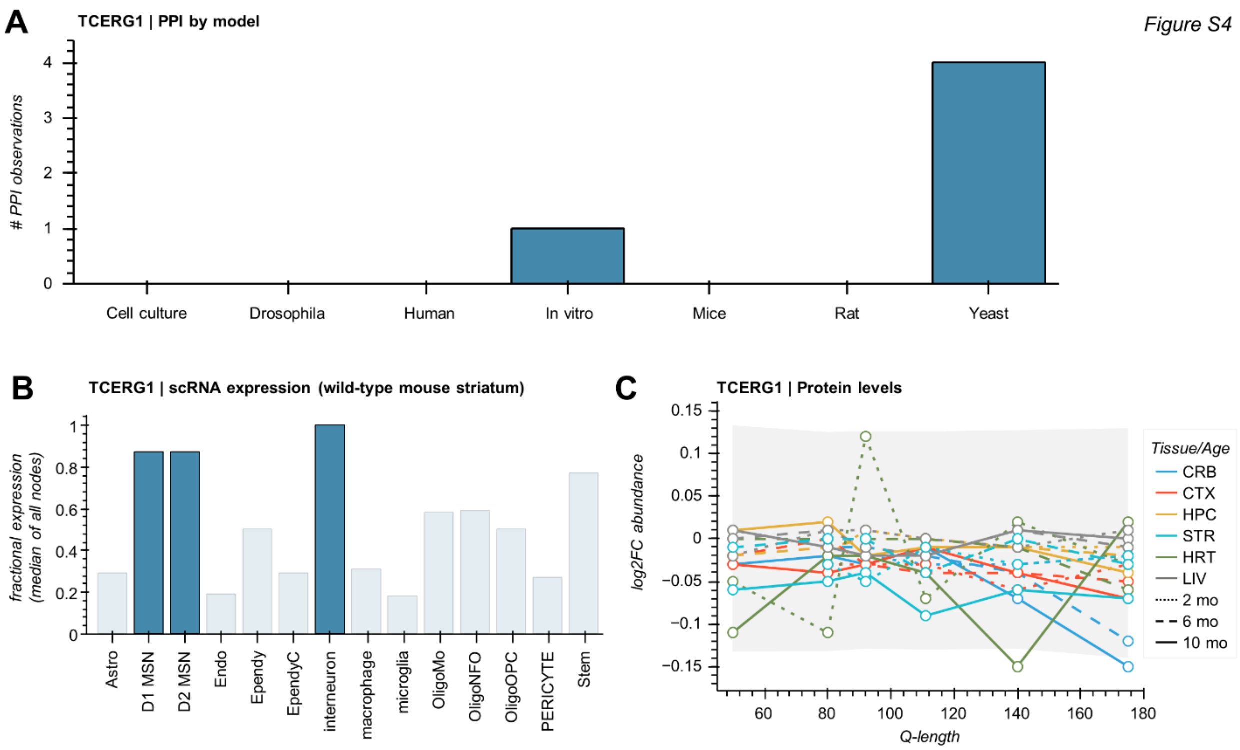

Figure S4: TCERG1 omics data, related to Figure 4

A) TCERG1 PPI observations across different experimental models. B) TCERG1 scRNA expression levels across different striatal cell subtypes in wild-type (WT) mouse striatum. Dark blue bars are those with values ≥ 0.8, representing particularly high expression levels. C) TCERG1 protein levels across different mouse tissues, ages, and Q-lengths. Grey shaded boxes represent a bounding box for 90% of the underlying data (i.e., if a datapoint is outside of these bounds, it is in the upper or lower 5% of the data at that Q-length); CRB = cerebellum, CTX = cortex, HPC = hippocampus, STR = striatum, HRT = heart, LIV = liver.

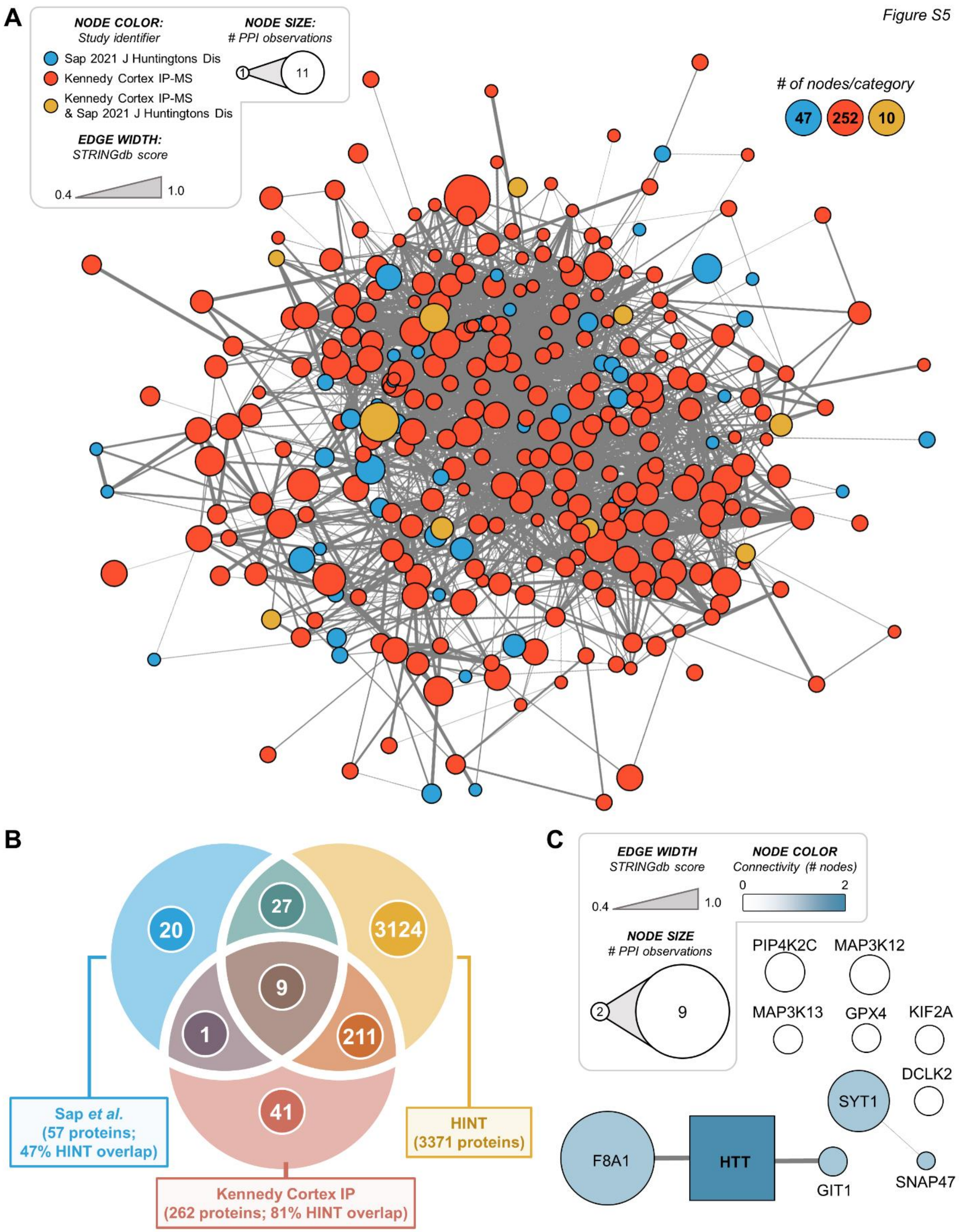

Figure S5
A
NODE COLOR:
Study identifier
Sap 2021 J Huntingtons Dis
Kennedy Cortex IP-MS
Kennedy Cortex IP-MS & Sap 2021 J Huntingtons Dis
NODE SIZE:
PPI observations
1
11
EDGE WIDTH:
STRINGdb score
0.4
1.0
# of nodes/category
47
252
10
B
20
27
3124
9
1
211
41
Sap et al. (57 proteins; 47% HINT overlap)
HINT (3371 proteins)
Kennedy Cortex IP (262 proteins; 81% HINT overlap)
C
EDGE WIDTH
STRINGdb score
0.4
1.0
NODE COLOR
Connectivity (# nodes)
0
2
NODE SIZE
PPI observations
2
9
PIP4K2C
MAP3K12
MAP3K13
GPX4
KIF2A
SYT1
DCLK2
F8A1
HTT
GIT1
SNAP47

Figure S5: Kennedy Cortex IP-MS overlap with Sap *et al.* 2021 cortex XL-IP-MS, related to Figure 5

A) Shared and unique HIPs between the cortex IP-MS dataset generated by this study (Kennedy Cortex IP-MS) compared to the crosslinking cortex IP-MS study published in Sap *et al.* 2021. Nodes are colored by whether they are unique or shared between the two datasets. B) Venn diagrams depicting the overlap between the Kennedy Cortex IP-MS dataset, the Sap *et al.* dataset, and the HINT database. C) HTT-OMNI network of 10 shared HIPs identified in both the Kennedy Cortex IP-MS and Sap *et al.* studies. Nodes are colored by their relative connectivity to one another. Edges are defined for HTT PPIs that have known STRINGdb functional interactions.

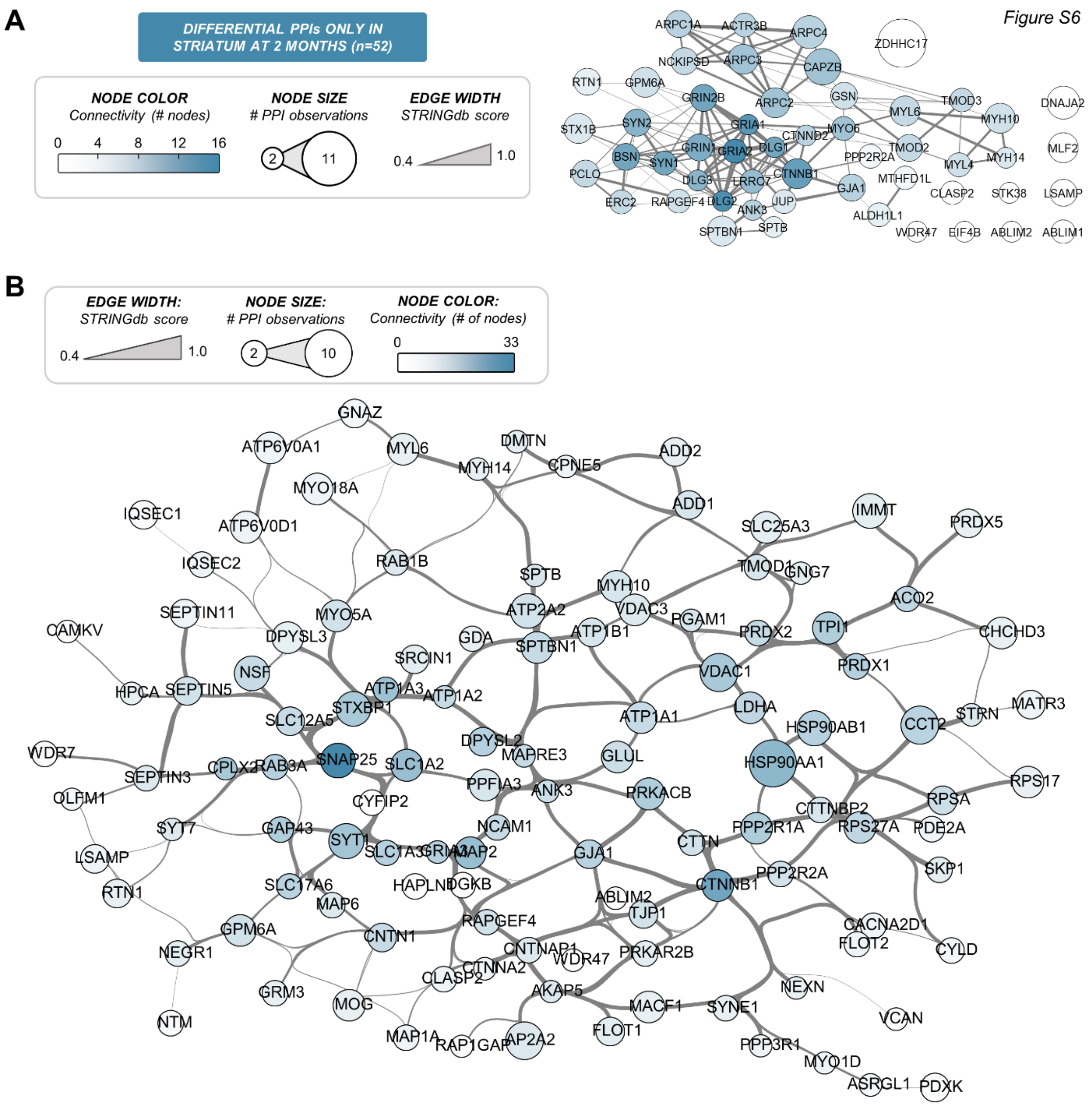

Figure S6: HTT-OMNI network of differential or stable and specific HIPs only in the striatum, related to Figure 6 and 8

A) Network of HTT interactions that are polyQ-dependent in the striatum by 2 months of age and unchanged in cortex at both ages. B) Network of stable and specific HIPs only in the striatum. The network was assembled in HTT-OMNI by uploading Table S6 (corresponding to the network in Fig. 5C) and filtering by human gene symbols obtained from the set of specific and stable HIPs unique to the striatum (n = 23+89+7 in Fig. 8D).
